# Synthesis and photophysical investigations of pyridine-pyrazolate bound boron(III) diaryl complexes

**DOI:** 10.1038/s41598-022-20796-2

**Published:** 2022-10-01

**Authors:** Rashid Javaid, Aziz Ul Rehman, Manan Ahmed, Mohammad Hashemi Karouei, Nima Sayyadi

**Affiliations:** 1 Department of Molecular Sciences, Macquarie University, Sydney, NSW 2109 Australia; 2 ARC Centre of Excellence for Nanoscale Bio Photonics (CNBP), Macquarie University, Sydney, NSW 2109 Australia; 3 Agriculture & Biophotonics Division, National Institute of Lasers and Optronics College, Pakistan Institute of Engineering and Applied Sciences (PIEAS), Islamabad, 45650 Pakistan; 4 School of Chemistry, The University of New South Wales, Sydney, 2052 Australia

**Keywords:** Coordination chemistry, Inorganic chemistry, Photochemistry

## Abstract

This study presents the design and synthetic pathway of unsymmetric ligands based on pyridine-pyrazolate scaffold with Donor–Acceptor (D–A) molecular arrays and their boron complexes to achieve a large Stokes shift. Intermolecular charge transfer (ICT) triggered by the uneven molecular charge distribution from electronically dense pyrazolate (donor) part of the ligands to electron-deficient boron centre (acceptor) resulted in a mega Stokes shift up to 263 nm for selected compounds while retaining the characteristic quantum efficiency and chemical stability. The photophysical properties of derivatization of pyrazolate group in the pyridine-pyrazolate scaffold of diaryl boron complexes were explored based on UV–Visible, steady-state and time-resolved fluorescence spectroscopy. An interesting dual emission along with quenching behaviour was also observed for 2-(6-methoxynaphthelene) 5-(2-pyridyl) pyrazolate boron complex (P_5_) due to the formation of a twisted intermolecular charge transfer (TICT) state from a locally excited (LE) state rendering it a potential candidate for sensing applications based on H-Bond quenching. In addition, the extended excited state lifetime of the reported compounds compared to classical boron-dipyrromethene (BODIPY) makes them suitable as potential probes for analytical applications requiring a longer excited state lifetime.

## Introduction

Small organic and organometallic fluorescent dyes are vital in the modern world and have applications in a variety of domains such as nanoscience^1^, solar energy conversion^2^, and biological chemistry^3,4^. Attributes of a successful dye include high absorption coefficient and quantum yields, large Stokes shifts, tunability and high photo and chemical stability^5^. In this regard, organoboron complexes enjoy a great deal of attention due to their unique fluorescence properties and high levels of structural diversity which can be managed by changing the organic ligands involved in coordination^6,7^. Among various types of boron complexes, boron dipyrromethene (BODIPY) are well-known and one of the main fluorescent dye with some excellent properties. For instance, their high fluorescence quantum yields, sharp absorption/emission spectra, tunability, ease of functionalization^8^ and chemical stability^9–12^. In addition, the properties of BODIPY have been also found in photodynamic therapy agents^13,14^, chemosensors^15,16^, solar cells^17,18^ and potential to be used as thermal and thermoelectric devices owing to their exceptional thermoelectric effect^19,20^. However, most BODIPY dyes and their derivatives have certain disadvantages such as small Stokes shifts (typically 5–20 nm) due to their rigid structure which are usually responsible for self-quenching and background scattering of its own fluorescent. Secondly, the BODIPY dyes are commonly faint or non-fluorescent in the solid state due to the strong π–π stacking of planar fluorophores^21^. Thus, this deficiency limits their use as a material for comprehensive applications such as biological imaging^22^.

A straightforward approach to overcome this problem is the rational design of boron fluorescent dyes with low-symmetry or asymmetric *N,N*-ligands, in particular, desymmetrisation of the standard BODIPY core with a donor–acceptor (D–A) architecture^23^. Notable examples are ketoiminosoindolines, benzothiazole-pyrimidines, carbazole-benzimidazoles, carbazole 2-azoles, benzothiazole-pyrimidines, and pyridyl-enamido-based derivatives. Such an approach has been reported to have not only resulted in large Stokes shifts and solvatochromism but also high luminescence efficiency, the mechanism for the phenomenon was visualized using charge density difference (CDD) recently^24–26^. This is because of the two-photon scaffolds nature of these molecules and their strong intramolecular charge transfer (ICT) in excited states, as a result, their emission wavelength exhibits a large redshift with respect to their environment such as a change in polarity of solvents. However, it is also noted that under a polar environment (physiological condition) D-A type architecture suffers serious twisted internal charge transfer (TICT) and external conversion (EC) which produces severe fluorescence quenching, as a result, high single-to-background ration (SBR)^27–31^.

Recently, the synthesis of N-B-N and N-B-O based boron complexes using heterocycle and imido or imine nitrogen or enolate/phenolate oxygen or between two different heterocyclic systems are rapidly growing areas in this field. Because such complexes are a promising candidate for a range of possibilities in terms of structural variation and exhibit a large Stroke shift^32–35^. For instance, Chi and co-workers have previously reported the synthesis of boron compounds based on the pyridine-pyrazolate ligands scaffold by adopting a pull–push approach and observed interesting solvent-dependent properties^36^. However, herein a distinct methodology was adopted to execute efficient fluorescent boron complexes based on pyridine-pyrazolate ligands with a D-A architecture through the gradual increase of electron density on the derivatized pyrazolate (donor) side of the ligands with the pyridyl site acting as the acceptor using literature protocols^37^. The effect of varying electronic density on the ligand scaffold through the inclusion of different functional groups is studied by UV–Visible (UV–Vis) absorption, excitation/ emission, and excited-state lifetimes of the resulting fluorophore in different solvents.

Bearing in mind the importance of fluorescent dyes, herein we have designed and synthesised a new family of boron complexes based on pyridine-pyrazolate ligands with improved features including large Stokes shift, high emission efficiency, extended excited-state lifetime and solvatochromism, compared to classical BODIPY dyes.

## Results and discussion

### Synthesis and characterization

**Figure 1:**
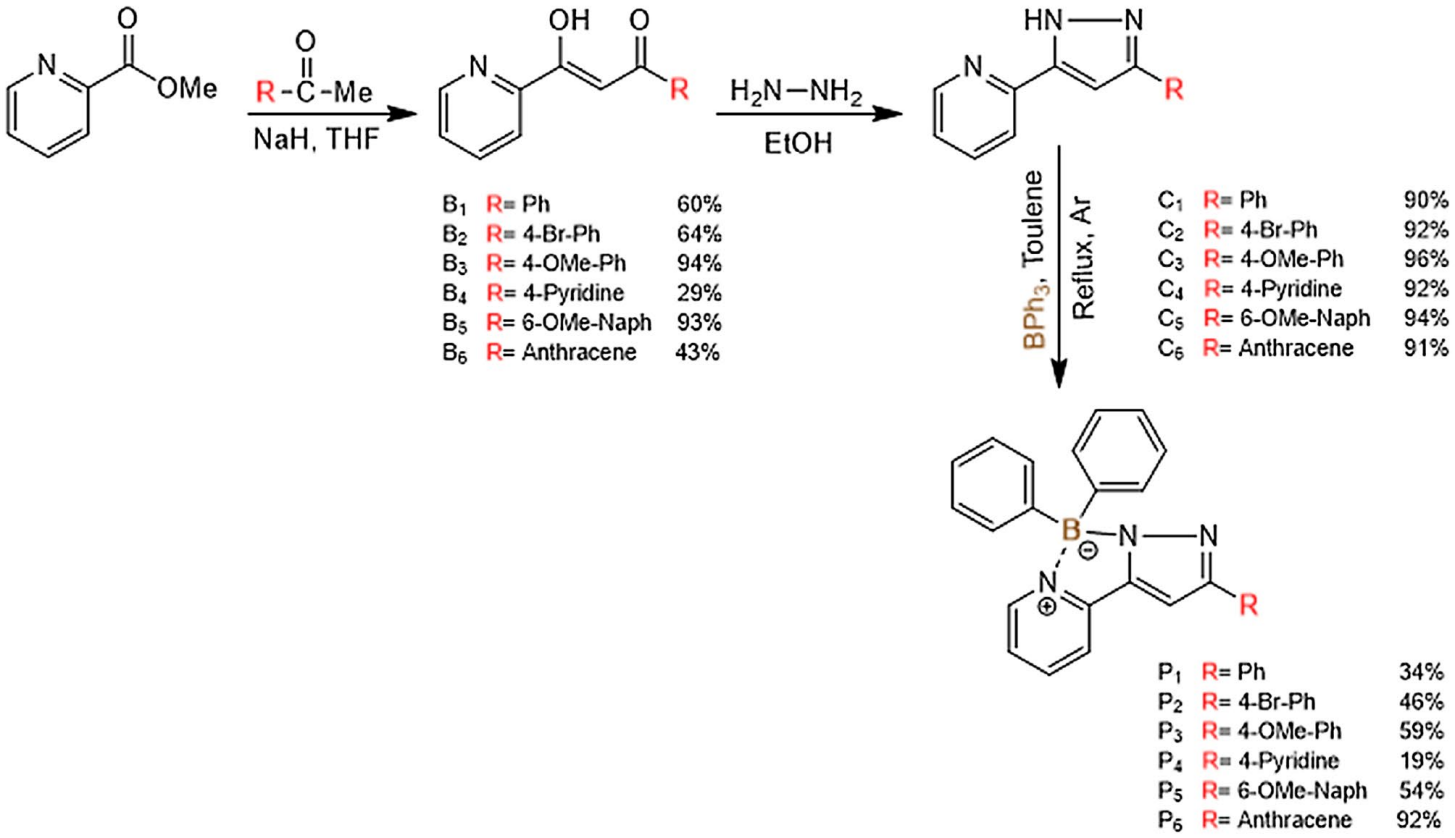
Synthetic route for the pyridine-pyrazolate boron complexes.

The three-step synthetic route used in this work for the preparation of the pyridine-pyrazolate ligands scaffold and their biphenyl boron complexes is summarized in Fig. 1. P_1_–P_6_ were synthesised utilizing optimized conditions^38^ via a 1,3-diketone pathway using Claisen condensation^36^. The methyl ester of picolinic acid was refluxed with the corresponding ketone using sodium hydride (NaH) as a catalyst. Tetrahydrofuran (THF) was dried prior, as the synthesis was found to be moisture sensitive^1,39^^1^H nuclear magnetic resonance (NMR) analysis of 1,3-diketones (B_1_-B_6_) inferred that they exist in keto-enolic tautomeric forms, the enolic form was found to be dominant (a distinct enolic proton at around 16 ppm of enolic and methine (CH) as singlet). Pyridine-pyrazolate ligands were prepared by refluxing 1,3-diketones with hydrazine hydrate. The reaction was efficient with yields ranging from 90 to 100%. Finally, the ligands were coordinated by refluxing with BPh_3_ in toluene until, they were consumed completely, as monitored by thin layer chromatography (TLC) analysis. The resulting boron complexes were isolated using column chromatography (silica gel) as white (P_1_, P_2_, P_4_, P_5_) and yellow (P_3_, P_6_) crystalline solids in yields ranging from 19 to 92%.

The preliminary confirmation of product formation was made by comparing the ^1^H NMR of the product with that of the precursor 1,3 diketone (B_1_–B_6_) through the characteristic protons of the diketone *i.e.,* enolic and methine. Upon pyrazole ring (C_1_–C_6_) formation the enolic proton disappeared, and a new broad peak appeared at around 12 ppm indicative of the –NH functional group. Further shifting of ^1^H resonance peaks up field, consistent with the replacement of the electronegative oxygen with comparatively less electronegative nitrogen was observed. The chelation of the ligand with boron was marked by the disappearance of –NH and further up field shift in the proton resonance signals upon formation of the complex. The purity of the final compounds was further confirmed by ^13^C NMR and high-resolution mass spectroscopy (HRMS) analysis (ESI-appendix).

The boron complexes displayed stability towards the air, moisture and room temperature for extended periods and no changes were observed based on the NMR and UV–Vis analysis. All the complexes were soluble in organic solvents including dichloromethane (DCM), THF, ethyl acetate (EA), chloroform, methanol, ethanol, acetone, benzene, pyridine, and toluene. P_1_–P_4_ have been reported elsewhere for their usefulness as Raman reporter molecules^40^.

### Photophysical properties

**Figure 2:**
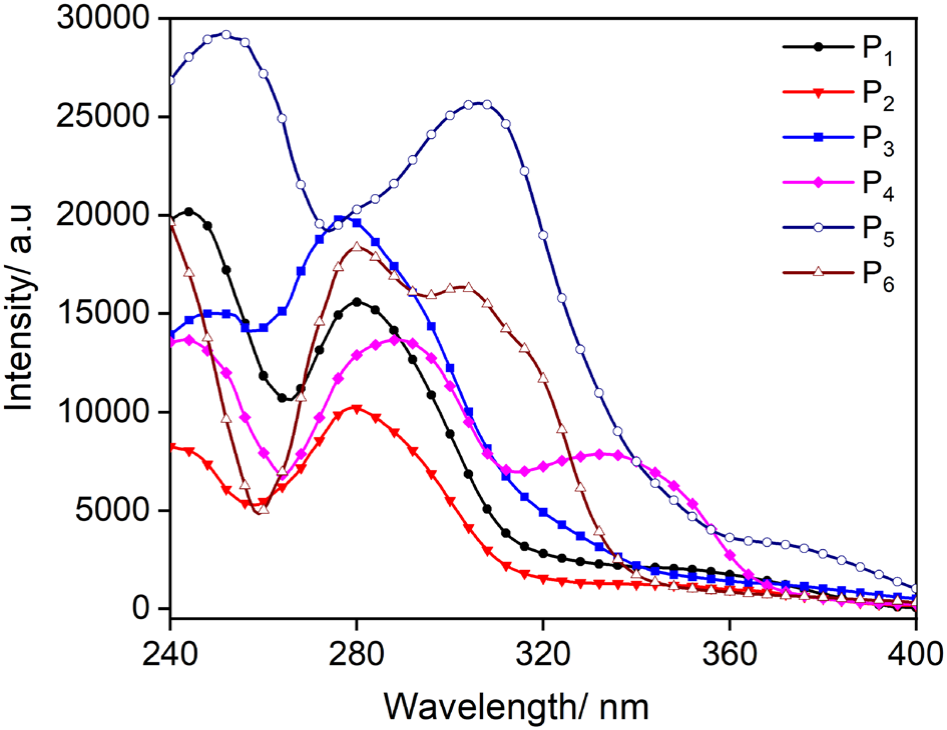
UV–Vis absorption spectra of P_1_–P_6_ in CH_2_Cl_2_ (25 µM) at 298 K.

To evaluate the potential of the synthesised dyes as fluorescent probes, UV–Vis absorption and fluorescence studies were carried out in CH_2_Cl_2_ at a concentration of 25 µM and 298 K, depicted in (Fig. 2) and summarized in Table 1. The parent compound P_1_ exhibited two distinct transitions, a higher energy transition observed at 244 nm and a moderately strong peak at slightly lower energy around 280 nm with a higher molar absorption coefficient (Ɛ = 20,300 M^−1^ cm^−1^). The transitions can be tentatively assigned to the planar pyridyl pyrazolate ligands where the highest occupied molecular orbital (HOMO) mainly resides on pyrazolate and the lowest unoccupied molecular orbital (LUMO) on the pyridine moiety^36,38^. The absorption profiles do not vary significantly for complexes P_1_–P_3_ and P_5_ due to their similar framework, however, P_4_ and P_6_ displayed different absorption behaviour having two slightly red-shifted bands (P_4_ = 249, 288 nm, P_6_ = 246, 280 nm) compared to the parent complex, and also an additional third band at 335 nm and 310 nm, respectively due to the presence of pyridine and anthracene in the ligand scaffold^41,42^. While, all compounds displayed a negligible substitution effect however no regular trend was observed. Similar anomalous behaviour was observed in *Ɛ* values and the reason for the shifting of the *Ɛ* values is unclear^43–45^.

**Table 1:** Photophysical properties of synthesized boron complexes.

Absorption (CH_2_Cl_2_)			Emission				
Compound	λ_abs_[nm](ε[M^−1^ cm^−1^])^a^	$\tau$_o_ (ns) ^a, b^	λ_em[nm]_^a^	Stokes Shift [nm] ^a^	Φ_F_^a^	K_nr[ns]_^c^	K_r[ns]_^c^
P_1_	244 (20,300)	12.94	463	219	0.50	0.039	0.039
P_2_	278 (10,200)	6.96	461	183	0.64	0.052	0.091
P_3_	277 (19,900)	10.43	508	231	0.71	0.028	0.068
P_4_	288 (13,700)	8.83	433	145	0.62	0.044	0.07
P_5_	250 (29,400)	10.62	370,513	120, 263	0.43	0.054	0.04
P_6_	280 (18,400)	10.05	486	206	0.71	0.029	0.071

^a^λ_abs (_absorption)_,_ λ_em_ (emission) measured in CH_2_Cl_2_ at 25 µM and room temperature. Uncertainty for λ_abs_ and λ_em_: ± 1 nm. Uncertainty for $\tau$_o_ ± 0.3 ns.

^b^Lifetimes measured using CH_2_Cl_2_ under an inert atmosphere. Uncertainty for $\tau$_o_: ± 0.1 ns.

^c^Rates constants of radiative (k_r_) and non-radiative (k_nr_) decay calculated using the formula $K_{r} = \frac{\Phi F}{{\tau o }}$ and (Φ_F_ stands for emission efficiency) $\tau$_o_ refers to lifetime. ${\text{K}}_{{{\text{nr}}}} = \frac{{\left( {1 - \Phi F } \right)}}{\tau o}$.

**Figure 3:**
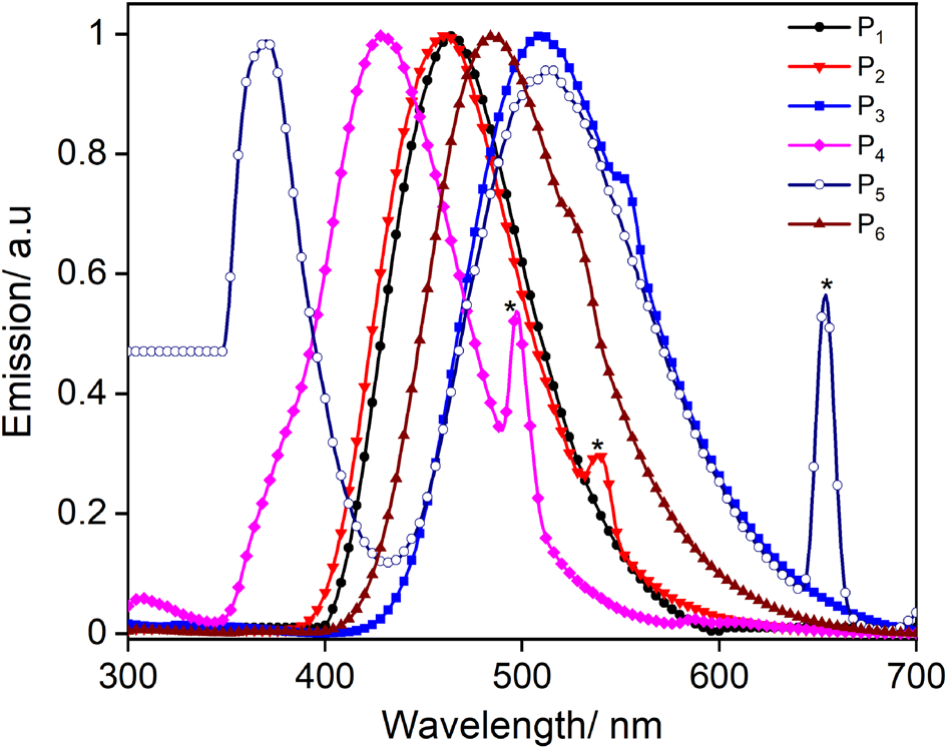
Normalized emission spectra of P_1_–P_6_ in CH_2_Cl_2_ (25 µM) at 298 K. Scattering is marked as *.

The emission profiles observed for all synthesised compounds (P_1_–P_6_) ranged from violet to green region of the electromagnetic spectrum (428 nm to 513 nm) Fig. 3. The emission maxima of 463 nm for P_1_ were found in accordance with the previously reported emission values for the compound^36,38^. The effect of substitution in the emission profiles was more prominent compared to the behaviour observed in the UV–Vis absorption spectra, as negligible changes for P_2_ were observed compared to the parent compound, while P_3_ and P_5_ displayed a significant red shift due to electron-donating nature of –OMe group. A dual emission behaviour was observed for P_5_. All the tested compounds displayed larger Stokes shifts ranging from 143 to 263 nm, than that typically found in the case of BODIPY’s (5–20 nm)^9^ (Fig. 3).

Large Stokes shifts are usually dependent on solvent re-orientation and excited state conformational changes *i.e.,* the geometry differences between the ground state (S_0_) and the energy-minimized first excited state (S_1_) or both^28,46,47^. In principle, unsymmetrical fluorescent compounds exhibit energetically distinct S_0_ and S_1_ states and ICT contributes to the large Stokes shift^48^. The nearly planar architecture of the synthesised compounds and their unsymmetric nature are potentially responsible for the emergence of large Stokes shifts, with pyrazolate acting as an electron donor to electron-deficient pyridyl centre facilitating ICT. The relative photoluminescence quantum yields (PLQY) of these compounds were measured using quinine hemisulfate monohydrate as a standard. All the compounds show high quantum yields with P_3_ and P_6_ displaying the highest value of 0.71. The lowest emission efficiency and highest non-radiative decay were observed for P_5_ (Table 1).

Further insight into the fluorescence properties was obtained by time-resolved fluorescence spectroscopy. All the excited-state lifetimes displayed monoexponentially decay with lifetimes ranging from 6.96 to 12.94 ns in DCM compared to the classical BODIPY dyes having a lifetime of 2 ns^9^. All tested compounds except P_5_ displayed no effect of solvents on their photophysical properties.

### Solvatochromic behavior of P_5_

**Figure 4:**
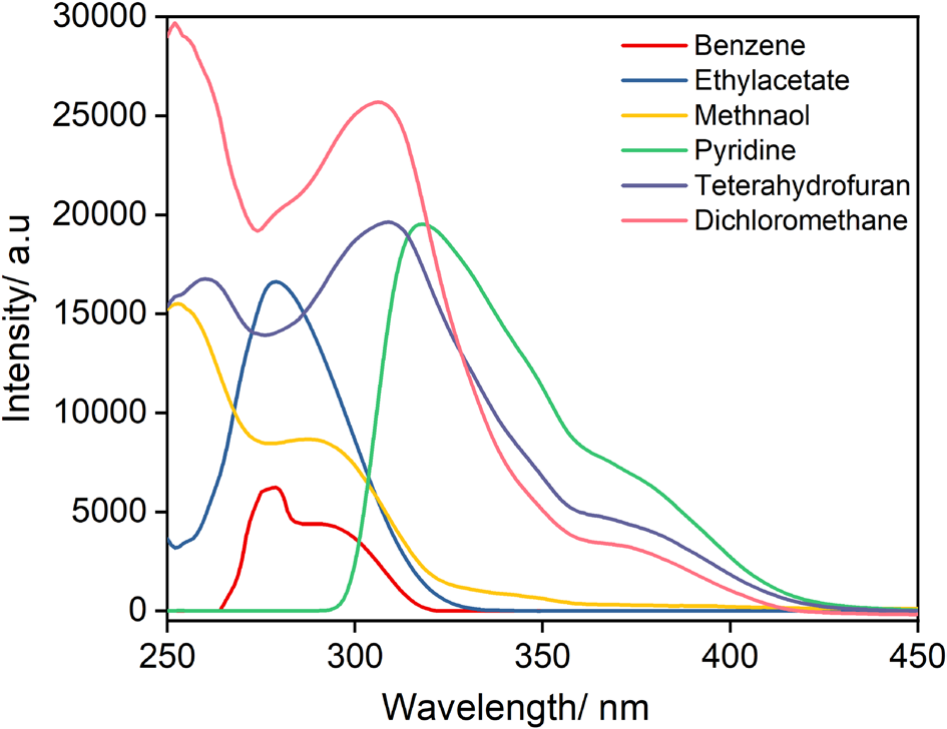
UV–Vis absorption spectra of P_5_ (25 µM) as a function of solvent s at 298 K. Spectra zeroed at 450 nm, even for compounds with significant scattering apparent*.*

**Figure 5:**
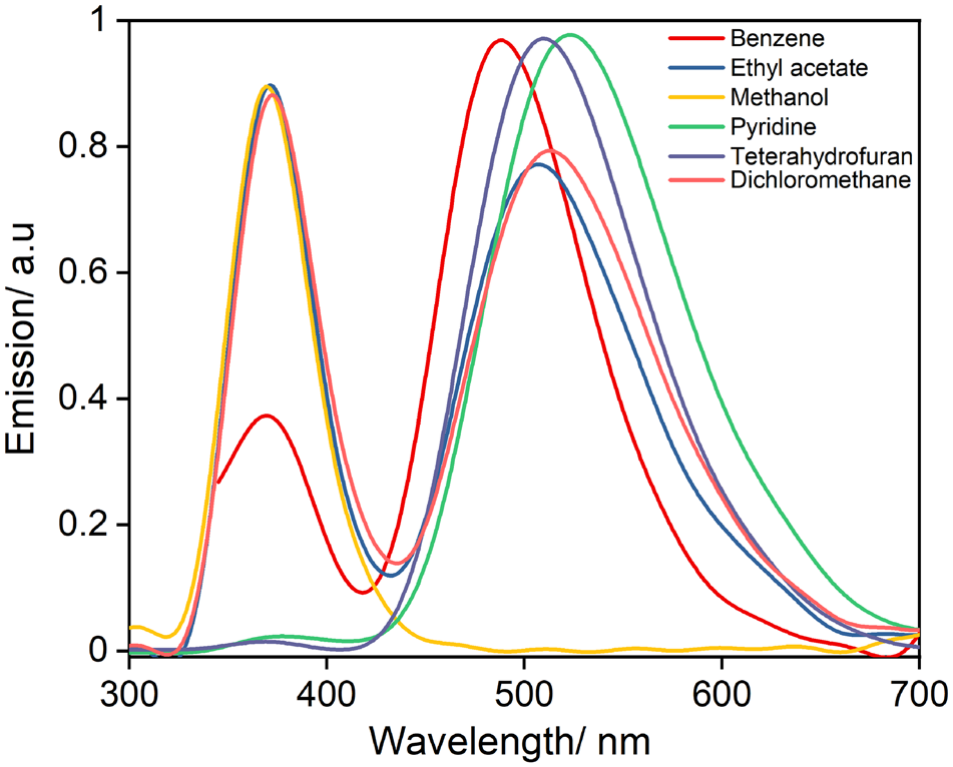
Normalized emission spectra of P_5_ (25 µM) as a function of solvent at 298 K.

The absorption and emission maxima of P_5_ exhibited pronounced shifts as a function of solvent polarity (Figs. 4, 5 and Table 2). The absorption maxima shifted significantly as the solvent polarity was increased, implying that the ground state is also affected by the solvent polarity possibly due to a change in the dipole moment of the dye in the ground state^49,50^. A shift of 65 nm was found between methanol to pyridine. Similar shifts were obtained in the case of fluorescence along with the dual emission behaviour.

**Table 2:** Solvatochromic properties of P_5._

Absorption (CH_2_Cl_2_)			Emission			
Solvent	λ_abs[nm]_(ε[M^−1^ cm^−1^])^a^	$\tau$_o_ (ns) ^a,b^	λ_em[nm]_^a^	Φ_F_^a^	K_nr[ns]_^c^	K_r[ns]_^c^
Benzene	278(6200)	6.83	371,488	0.73	0.04	0.106
Ethyl Acetate	278(16,700)	5.64	371,508	0.68	0.056	0.121
THF	308(19,600)	6.19	508	0.90	0.017	0.145
DCM	250(29,400)	10.62	370,513	0.43	0.054	0.04
Pyridine	317(19,600)	2.90	524	0.95	0.019	0.326
Methanol	252(15,700)	3.83	371	0.37	0.164	0.097

^a^Measured in CH_2_Cl_2_ at 25 µM and room temperature. Uncertainty for λ_abs_ and λ_em_: ± 1 nm. Uncertainty for $\tau$_o_ ± 0.3 ns.

^b^Lifetimes measured using CH_2_Cl_2_ under an inert atmosphere. Uncertainty for $\tau$_o_: ± 0.1 ns.

^c^Rates constants of radiative (k_r_) and non-radiative (k_nr_) decay calculated using the formula ${\text{K}}_{{\text{r}}} = \frac{\Phi F}{{\tau o }}$ and ${\text{K}}_{{{\text{nr}}}} = \frac{{\left( {1 - \Phi F } \right)}}{\tau o}$.

Previously reported similar compounds^36^ were found to show dual emission and solvatochromism having one band, F_1_, at around 370 nm that was independent of the solvent but a second band, F_2_, at a longer wavelength which was solvent sensitive. Interestingly, both emission bands were found to be solvent responsive for P_5_ in specific organic solvents (Fig. 5). For instance, in the case of THF and pyridine, while the F_1_ band disappeared, the second emission band (F_2_) was observed with maxima at 508 and 524 nm, respectively. Another interesting solvent-dependent feature is the quenching by solvents capable of hydrogen bonding, presumably due to a form of hydrogen-bonded charge transfer^51^. For protic solvents, strong quenching and shorter lifetime are due to the additional interactions that are common for protic solvents. This feature can be utilized to probe the protic solvents as well for the complex biological systems^52^.

Although the origin of dual fluorescence is still not clear, there are various hypotheses to explain the phenomenon^53^. Fluorophores with D-A configuration upon absorption of a photon, undergo an internal proton transfer from a donor part of the molecule to an acceptor that results in a relaxed perpendicular conformer which exists in equilibrium with the coplanar conformer. These geometry changes result in dual emission, where a narrow high energy band is the result of local excitation (LE = 370 nm) and a lower energy broader band emerges due to TICT^54^. In the TICT state, the donor part is highly twisted with respect to the acceptor part of the complex resulting in charge separation, increasing the solvent polarity pushes the equilibrium favouring the TICT that potentially accounts for the disappearance of the LE band when using THF and pyridine. It is noteworthy this behaviour was only observed with specific polar solvents having donor atoms, hence complex-solvent interactions need to be considered^55^.

The excited-state lifetimes revealed that except in the case of methanol and DCM, higher radiative rates and higher emission efficiency are favoured as can be seen in Table 2. Particularly, solvents with donor atoms exhibited a special effect on the quantum yields (PLQY) as well, as their values reached up to 0.95 for pyridine and 0.90 for THF. While a very short excited state lifetime confirmed their origin to be TICT. Furthermore, strong quenching and shortening of the lifetime were noticed for typical protic solvents, owing to hydrogen bonding interactions.

## Conclusions

A new series of diphenyl boron (III) complexes bearing unsymmetric pyridine-pyrazolate ligands with donor–acceptor (D–A) features has been synthesised and characterized using ^1^H, ^13^C NMR and HRMS analysis. The complexes exhibited tunable emission, high luminescence efficiency and large Stokes shift properties depending on the electronic nature of the functional groups on the ligands. The longer Stokes shift along with longer lifetime compared to classical BODIPYs renders them strong candidates for applications as probes requiring a longer life with minimal self-quenching and background scattering. Selected complexes (P_5_) displayed dual emission behaviour and interesting solvatochromic behaviour. Owing to the quenching behaviour observed for complexes in H-bonding solvents, this complex could be potentially useful in sensing applications for the detection of a biologically and environmentally interesting analyte. The future investigation includes the modification of synthesised compounds by cleaving –OMe to –OH to tag various antibodies for analysis. Secondly, the –Br functional group provides a potential centre for further modifications and tuning to obtain dyes that can potentially emit in the NIR region.

## Experimental

### Methods

NMR and HRMS graphs are reported in the supporting information. Where otherwise noted, all chemicals purchased were from a commercial supplier and were used without further purification. The THF solvent used for the synthesis of 1,3-diketones were first dried with a molecular sieve 4 Å and was stored under a nitrogen atmosphere for 72 h before being used. All the reactions were carried out under an inert atmosphere of argon. The progress of the reaction was monitored by TLC analysis. High-resolution mass spectra were recorded with a mass spectrometer (Agilent 6538 Q-TOF with dual ESI source). ^1^H and ^13^C NMR spectra were recorded on a Bruker Advance spectrometer [400 MHz (^1^H) and 100 MHz (^13^C) in CDCl_3_ (first de-acidified by passing it through calcium carbonate before testing) and with DMSO. The solvent peaks were referenced according to the literature^56^. UV–visible absorption spectra were recorded on Eppendorf UV–Vis spectrophotometer. The fluorescence spectra were measured on Varian carry fluorescence spectrophotometer. The fluorescence lifetime was determined using Fluoromax-4 fluorimeter (Horiba) in a quartz cuvette (Starna). Excitation was carried out using a 293-nm delta diode (Horiba) in Fluoromax-4C-TCSPC configuration. The diode was pulsed at a 2 MHz repetition rate, the decay was measured until 10,000 counts were reached in the peak channel.

### Materials

2-Picolinic acid (98-98-6), sulphur acid (7664-93-9), sodium bicarbonate (144-55-8), sodium hydride (7646-69-7), 6-methoxy 2-acetonaphthone (3900-45-6), 4-bromo acetophenone (99-90-1), acetophenone (98-86-2), 4-methoxy acetophenone (100-06-1), 4-acetylpyridine (1122-54-9), 2-acetyl anthracene (784-04-3), magnesium sulfate (7487-88-9), hydrazine hydrate (10,217-52-4), triphenyl borane (960-71-4), quinine hemisulfate salt monohydrate (207,671-44-1), sodium chloride (7647-14-5), silica (112,926-00-8), molecular sieves 4A^0^ (20,300), methanol (67-56-1), dichloromethane (75-09-2), tetrahydrofuran (109-99-9), Pyridine (110-86-1), chloroform (67-66-3), ethanol (64-17-5), chloroform-d (865-49-6), DMSO-d_6_ (2206-27-1) were purchased from Sigma Aldrich, Australia. Methyl picolinate used for the synthesis of 1,3-diketones was synthesized according to a reported method^57^.

### Synthesis of 1, 3-diketone

#### General procedure

Sodium hydride (5 equivalent) was suspended in dry THF (40 mL) in an ice bath. To this methyl picolinate (2.5 equivalent), suitable acetophenone (1 equivalent) was added under an atmosphere of argon. The resulting mixture was refluxed overnight under an atmosphere of argon. After cooling the reaction mixture to room temperature, it was poured into ice and neutralized with acetic acid (2 mL). The crude material was extracted with DCM (3 × 30 mL). The combined organic layers were washed with brine (2 × 50 mL), dried over magnesium sulfate, filtered, and evaporated to afford crude material. This was further purified by column chromatography on silica gel with a suitable combination of solvents as eluent.

#### 1-phenyl-3-(pyridine-2-yl) propane-1, 3-dione (B_1_)

Sodium hydride (50 mg, 20.4 mmol), methyl picolinateA_1_ (2.85 g, 20.8 mmol) and acetophenone (1.00 g, 8.3 mmol) was used. The crude material was purified using column chromatography (silica, DCM*, n*-hexane 1:1) to afford **B**_**1**_ (1.13 g, 60%) as a yellow solid. ^1^H NMR (400 MHz, CDCl_3_) 7.44–7.58 (m, 5H, ArH), 7.83–7.88 (m, 1H, ArH), 8.06–8.09 (m, 2H, ArH), 8.15–8.17 (m, 1H, ArH), 8.70–8.72 (m, 1H, ArH), 16.47 (s, 1H, OH enolic) ppm. ^13^C NMR (100 MHz, CDCl_3_) 93.69, 122.26, 126.48, 127.57, 127.62, 128.77, 128.82, 132.78, 135.45, 137.17, 149.41, 152.68, 183.64, 186.42 ppm. HRMS (ESI, TOF) m/z calcd for C_14_H_12_NO_2_, 226.0868; found 226.08606, the data are in agreement with the reported literature^58^.

#### 1-(4-bromophenyl)-3-(pyridine-2-yl) propane-1, 3-dione (B_2_)

Sodium hydride (30 mg, 20.4 mmol), methyl picolinateA_1_ (1.72 g, 12.6 mmol) and 4-bromo acetophenone (1.00 g, 5.0 mmol) was used. The crude material was purified using column chromatography (silica gel, ethyl acetate*, n*-hexane 2:8) to afford **B**_**2**_ (98 mg, 64%) as a yellow solid. ^1^H NMR (400 MHz, CDCl_3_) 7.44–7.51 (m, 1H, ArH), 7.55 (s, 1H, CH, enolic), 7.63 (d, 2H, *J* = 8.52 Hz, ArH), 7.85–7.95 (m, 3H, ArH), 8.18 (d, 1H, *J* = 7.96 Hz, ArH), 16.35 (s, 1H, enolic, OH) ppm. ^13^ C NMR (100 MHz, CDCl_3_) 93.75, 122.34, 126.64, 127.62, 127.69, 128.77, 129.08, 132.07, 132.15, 134.33, 137.22, 149.43, 152.46, 184.14, 185.05 ppm. HRMS (ESI, TOF) m/z calcd for C_14_H_12_NO_2_ Br, 303.99731; found 303.99662.

#### 1-(4-methoxyphenyl)-3-(pyridine-2-yl) propane-1, 3-dione (B_3_)

Sodium hydride (40 mg, 16.7 mmol), methyl picolinateA_1_ (2.28 g, 16.7 mmol) and 4-methoxy acetophenone (1.00 g, 6.67 mmol) were used. The crude material was purified using column chromatography (silica gel, ethyl acetate*, n*-hexane 3:7) to afford **B**_**3**_ (1.59 g, 94%)^59^ as a bright yellow solid. ^1^H NMR (400 MHz, CDCl_3_) 3.89 (s, 3H, OCH_3_), 6.96–7.00 (m, 3H, ArH), 7.23–7.27 (m, 1H, ArH), 7.74–7.81 (m, 4H, ArH), 8.68 (d, 1H, *J* = 4.9 Hz, ArH),16.63 (s, 1H, –OH enolic) ppm. ^13^C NMR (100 MHz, CDCl_3_) 55.54, 93.60, 105.89, 119.82, 122.22, 124.31, 126.37, 127.26, 128.25, 128.87, 130.64, 131.20, 137.18, 137.29, 149.39, 152.78, 159.77, 182.78, 186.91 ppm. HRMS (ESI, TOF) m/z calcd for C_15_H_14_NO_3_, 256.09737; found 256.09653.

#### 1-(pyridine-2-yl)-3-(pyridine-4-yl) propane-1, 3-dione (B_4_)

Sodium hydride (49 mg, 20.7 mmol), methyl picolinateA_1_ (86 mg, 6.3 mmol) and 4-acetyl pyridine (1.00 g, 8.3 mmol) were used. The crude material was purified using column chromatography (silica gel, ethyl acetate*, n*-hexane 1:1) to afford **B**_**5**_ (55 mg, 29%) as bright yellow solid^60^ . ^1^H NMR (400 MHz, CDCl_3_) 7.41–7.44 (m, 2H, ArH), 7.80–7.88 (2H, ArH), 8.14 (d, 3H, *J* = 7.9 Hz, ArH), 8.73–8.74 (m, 2H, ArH), 15.94 (s, 1H, enolic –OH) ppm. ^13^C NMR (100 MHz, CDCl_3_) 94.67, 122.07, 122.24, 126.49, 127.30, 136.99, 137.03, 149.01, 149.63, 152.56, 184.56, 197.06 ppm. HRMS (ESI, TOF) m/z calcd for C_13_H_11_N_2_O_2_, 227.08205; found 227.08128.

#### 1-(6-methoxy naphthalene-2-yl)-3-(pyridine-2-yl) propane-1, 3-dione (B_5_)

Sodium hydride (15 mg, 6.3 mmol), methyl picolinateA_1_ (86 mg, 6.3 mmol) and 6-methoxy 2-acetonaphthone (50 mg, 2.5 mmol) were used. The crude material was purified using column chromatography (silica gel, ethyl acetate*, n*-hexane 1:9) to afford **B**_**4**_ (67 mg 93%) as bright yellow solid. ^1^H NMR (400 MHz, CDCl_3_) 3.88 (s, 3H, OCH_3_), 6.95–6.99 (m, 2H, ArH), 7.40–7.44 (m, 1H, ArH), 7.50 (s, 1H, C=CH), 7.85 (t,d 1H, *J* = 7.8 Hz, 1.8 Hz, ArH), 8.04–8.07 (m, 1H, ArH), 8.14 (d, 1H, *J* = 7.9 Hz, ArH), 8.69–8.71 (m, 1H, ArH), 16.62 (s, 1H, enolic –OH) ppm. ^13^C NMR (100 MHz, CDCl_3_) 55.43, 93.49, 105.79, 119.70, 122.11, 124.21, 126.24, 127.15, 128.15, 128.76, 130.55, 131.09, 137.07, 137.17, 149.27, 152.69, 159.66, 182.66, 186.81 ppm. HRMS (ESI, TOF) m/z calcd for C_19_H_16_NO_3_, 306.11302; found 306.11220.

#### 1-(anthracene-2-yl)-3-(pyridine-2-yl) propane-1, 3-dione (B_6_)

Sodium hydride (14 mg, 5.7 mmol), methyl picolinateA_1_ (78 mg, 5.7 mmol) and 3-acetyl anthracene (50 mg, 2.3 mmol) were used. The crude material was purified using column chromatography (silica gel, ethyl acetate*, n*-hexane 1:9) to afford **B**_**6**_ (55 mg, 74%). as a dull yellow solid.^1,61^ ^1^H NMR (400 MHz, CDCl_3_) 7.45–7.48 (m, 1H, ArH), 7.63–7.81 (m, 4H, ArH, C=CH), 7.84 (d, 1H, *J* = 8.9 Hz, ArH), 7.88–7.93 (m, 2H, ArH), 7.96 (d, 1H, *J* = 8.4 Hz, ArH), 8.20–8.25 (m, 2H, ArH), 8.76–8.78 (m, 1H, ArH), 8.85 (d, 1H, *J* = 8.2 Hz, ArH), 9.43 (s, 1H, ArH), 16.70 (s, 1H, enolic –OH) ppm. ^13^C NMR (100 MHz, CDCl_3_) 122.35, 122.98, 123.10, 123.41, 124.87, 126.48, 126.53, 127.31, 127.38, 128.86, 128.92, 129.04, 129.64, 130.15, 132.34, 133.15, 134.98, 137.23, 149.44, 152.79, 184.00, 186.33 ppm. HRMS (ESI, TOF) m/z calcd for C_22_H_16_NO_2_, 326.1181; found 326.11734.

### Synthesis of Pyridine-Pyrazole ligands

#### General procedure

Suitable 1, 3- diketone (1 equivalent) and hydrazine hydrate (3 equivalent) were dissolved in ethanol (30 mL) and heated to reflux overnight. The solvent was evaporated to dryness under reduced pressure to afford the respective pyridine-pyrazole ligands. The crude material was used without further purification.

#### 2-(3-phenyl-1H-pyrazol-5-yl) pyridine (C_1_)

Starting with B_1_ (50 mg, 2.2 mmol) and hydrazine hydrate (28 mg, 5.6 mmol), the crude C_1_ was obtained as a colourless solid (45 mg, 90%), which was used further without purification. ^1^H NMR (400 MHz, CDCl_3_) 7.11–7.13 (m, 1H, ArH), 7.26–7.33 (m, 1H, ArH), 7.35 (d, 1H, *J* = 6.9 Hz, ArH), 7.44 (t, 2H, *J* = 7.2 Hz, ArH), 7.79–7.86 (m, 4H, ArH), 8.66 (d, 1H, J = 4.9 Hz, ArH) ppm. ^13^C NMR (100 MHz, CDCl_3_) 100. 68, 120.37, 123.16, 125.82, 127.84, 128.24, 128.30, 128.60, 128.91, 132.53, 137.59, 144.64, 148.55, 149.18, 151.61 ppm. HRMS (ESI, TOF) m/z calcd for C_14_H_12_N_3_, 222.10312; found 222.10246.

#### 2-(3-(4-bromophenyl)-1H-pyrazol-5-yl) pyridine (C_2_)

Starting with B_2_ (40 mg, 1.3 mmol) and hydrazine hydrate (1.7 mg, 3.3 mmol), the crude C_2_ was obtained as a colourless solid (37 mg, 92%), which was used without further purification. ^1^H NMR (400 MHz, DMSO) 7.30–7.99 (m, 8H, ArH), 8.60 (s, 1H, ArH), 13.66, 13.56 (s, 1H, N= NH) ppm. ^13^C NMR (100 MHz, DMSO-*d*_6_) 100.78, 100.99, 101.18, 119.20, 119.99, 120.46, 121.24, 122.63, 123.07, 125.07, 127.07, 129.00, 131.57, 131.93, 132.76, 136.74, 137.36, 142.29, 143.31, 147.83, 149.17, 149.41, 150.12, 151.95, 152.32 ppm. HRMS (ESI, TOF) m/z calcd for C_14_H_11_BrN_3_, 300.01363; found 300.01290.

#### 2-(3-(4-methoxyphenyl)-1H-pyrazol-5-yl) pyridine (C_3_)

Starting with B_3_ (1.00 g, 3.9 mmol) and hydrazine hydrate (49 mg, 9.8 mmol), the crude C_3_ was obtained as a pink solid (98 mg, 100%), which was used without further purification. ^1^H NMR (400 MHz, CDCl_3_) 3.85 (s, 3H, OCH_3_), 6.96–7.00 (m, 3H, ArH), 7.23–7.27 (m, 1H, ArH), 7.74–7.81 (m, 4H, ArH), 8.68 (d, 1H, *J* = 4.9 Hz, ArH), 11.55 (s, 1H, NH) ppm. ^13^C NMR (100 MHz, CDCl_3_) 55.64, 93.02, 114.09, 122.11, 126.24, 128.25, 129.87, 137.16, 149.38, 152.76, 163.59, 181.71, 187.12 ppm. HRMS (ESI, TOF) m/z calcd for C_15_H_14_N_3_O, 252.11369; found 252.11289.

#### 2-(3-(pyridin-4-yl)-1H-pyrazol-5-yl) pyridine (C_4_)

Starting with B_4_ (30 mg, 1.3 mmol) and hydrazine hydrate (16 mg, 3.3 mmol), the crude C_4_ was obtained as a yellowish solid. (27 mg, 92%), which was used without further purification. ^1^H NMR (400 MHz, CDCl_3_) 7.24–7.27 (m, 3H, ArH), 7.41 (s, 1H, CH), 7.74–7.80 (m, 2H, ArH), 7.91 (d, 2H, *J* = 7.9 Hz, ArH), 8.66 (d, 2H, *J* = 4.4 Hz) ppm. ^13^C NMR (100 MHz, CDCl_3_) 101.97, 120.41, 121.94, 123.01, 137.21, 137.42, 148.21, 149.44, 150.13 ppm. HRMS (ESI, TOF) m/z calcd for C_13_H_11_N_4_, 223.09837; found 223.09772.

#### 2-(3-(6-methoxynaphthalen-2-yl)-1H-pyrazol-5-yl) pyridine (C_5_)

Starting with B_5_ (50 mg, 1.73 mmol) and hydrazine hydrate (21 mg, 4.3 mmol), the crude C_5_ was obtained as a yellowish solid (46 mg, 94%), which was used without further purification. ^1^H NMR (400 MHz, DMSO-*d*_6_) 3.89 (s, 3H, OCH_3_), 7.19 (d, 1H, *J* = 8.3 Hz, ArH), 7.35 (s, 3H, ArH), 7.85–7.98 (m, 5H, ArH), 8.30 (s, 1H, CH), 8.63 (s, 1H, ArH), 13.56 (s, 1H, NH) ppm. ^13^C NMR (100 MHz, DMSO-*d*_6_) 55.17, 55.22, 100.74, 100.81, 105.97, 118.85, 119.17, 119.32, 119.94, 122.52, 122.97, 123.35, 123.67, 123.87, 124.14, 124.37, 126.99, 127.45, 129.39, 128.57, 128.83, 129.47, 129.53, 133.75, 133.90, 136.69, 137.35, 143.15, 143.60, 148.02, 149.16, 149.41, 151.35, 152.18, 152.23 ppm. HRMS (ESI, TOF) m/z calcd for C_19_H_16_N_3_O, 302.12934; found 302.12852.

#### 2-(3-(anthracen-2-yl)-1H-pyrazol-5-yl) pyridine (C_6_)

Starting with B_6_ (40 mg, 1.2 mmol) and hydrazine hydrate (15 mg, 3.0 mmol), the crude C_6_ was obtained as a yellow solid (39 mg, 99%) which was used without further purification. ^1^H NMR (400 MHz, DMSO-*d*_6_) 7.30–8.24 (m, 11H, ArH), 8.65 (s, 1H, ArH), 9.03 (dd, 1H, *J* = 11.4 Hz, 7.30 Hz, ArH), 9.3 (s, 1H, apparent doublet due to H-bonding), 13.71 (s, 1H, N=NH), 13.76 (s, 1H, HN=N) ppm. ^13^C NMR (100 MHz, DMSO) 91.32, 96.06, 98.69, 101.27, 101.59, 102.01, 105.41, 106.06, 114.59, 118.95, 119.27, 119.99, 122.63, 123.05, 123.30, 123.98, 124.37, 126.58, 126.89, 127.11, 127.35, 128.54, 128.92, 129.26, 129.81, 130.04, 131.02, 131.15, 131.56, 131.88, 136.77, 137.40, 143.33, 143.60, 148.07, 149.21, 149.44 ppm. HRMS (ESI, TOF) m/z calcd for C_22_H_16_N_3_, 322.13442; found 322.13362.

### Synthesis of pyridine-pyrazole boron complexes

#### General procedure

Pyridine pyrazole ligand (1 equivalent) and triphenyl borane (1 equivalent) were dissolved in toluene (20 mL) and the resulting mixture was heated to reflux overnight under an atmosphere of argon. After the completion of the reaction was confirmed by TLC, the solvent was evaporated under reduced pressure. The residue was dissolved in DCM (40 mL) and the organic layer was washed with brine (50 mL), dried over magnesium sulfate, filtered and evaporated to obtain white and yellow material. The residue was purified using column chromatography on silica gel and a suitable combination of organic solvents.

#### 2-(5-phenyl-1H-pyrazol-3-yl) pyridine Boron Complex (P_1_)

Starting with C_1_ (7 mg, 0.3 mmol) and triphenyl borane (8 mg, 0.3 mmol) in toluene, the crude material was collected and purified using column chromatography (silica, ethyl acetate: *n*-hexane 1:1) to afford P_1_ as a white solid (108 mg, 34%). ^1^H NMR (400 MHz, CDCl_3_) 7.02 (s, 1H, ArH), 7.20–7.30 (m, 7H, ArH), 7.34–7.40 (m, 7H, ArH), 7.80 (d, t, 1H, *J* = 8.1 Hz, 1.0 Hz, ArH), 7.92–7.95 (m, 2H, ArH), 8.04 (d, t, 1H, *J* = 7.8 Hz, 1.1 Hz, ArH), 7.80 (d,t, 1H, *J* = 5.8 Hz, 1.0 Hz, ArH) ppm. ^13^C NMR (100 MHz, CDCl_3_) 97.52, 118.51, 121.85, 125.78, 126.07, 127.18, 127.52, 127.84, 128.07, 128.61, 128.92, 132.96, 134.40, 134.85, 141.59, 141.87, 143.28, 147.69, 157.56 ppm. HRMS (ESI, TOF) m/z calcd for C_26_H_20_BN_3_B, 385.18649; found 386.18240.

#### 2-(3-(4-bromophenyl)-1H-pyrazol-5-yl) pyridine Boron Complex (P_2_)

Starting with C_2_ (20 mg, 0.7 mmol) and triphenyl borane (16 mg, 0.7 mmol) in toluene, the crude material was collected and purified using column chromatography (silica, ethyl acetate: *n*-hexane 1:1) to afford P_2_ as a white solid (0.96 mg, 46%)**.** ^1^H NMR (400 MHz, DMSO-*d*_6_) 7.14–7.26 (m, 10H, ArH), 7.42 (s, 1H, C=CH), 7.59–7.61 (m, 2H, ArH), 7.68–7.72 (m, 1H, ArH), 7.82–7.85 (m, 2H, ArH), 8.25 (d, 1H, *J* = 8.0 Hz, ArH), 8.42 (t, d, 1H, *J* = 7.9 Hz, 1.4 Hz, ArH), 8.88 (d, 1H, *J* = 5.8 Hz, ArH) ppm. ^3^C NMR (100 MHz, DMSO-*d*_6_) 98.01, 119.24, 120.24, 123.59, 125.28, 126.69, 127.07, 127.42, 128.18, 128.87, 131.58, 132.26, 133.16, 137.31, 142.02, 143.67, 143.92, 145.76, 154.71 ppm. HRMS (ESI, TOF) m/z calcd for C_26_H_20_BBrN_3_, 464.0934; found 464.0941.

#### 2-(3-(4-methoxyphenyl)-1H-pyrazol-5-yl) pyridine Boron Complex (P_3_)

Starting with C_3_ (30 mg, 1.2 mmol) and triphenyl borane (29 mg, 1.2 mmol) in toluene, the crude material was collected and purified using column chromatography (silica, ethyl acetate: *n*-hexane 1:1) to afford P_3_ as a light-yellow solid (29 mg, 59%). ^1^H NMR (400 MHz, CDCl_3_) 3.82 (s, 3H, OCH_3_), 6.92–7.01 (m, 3H, ArH), 7.21–7.28 (m, 7H, ArH), 7.31–7.37 (m, 4H, ArH), 7.41–7.48 (m, 1H, ArH), 7.91–7.97 (m, 3H, ArH), 8.10–8.14 (m, 1H, ArH), 8.51 (d, 1H, *J* = 5.6 Hz, ArH) ppm. ^13^C NMR (100 MHz, CDCl_3_) 29.69, 55.30, 96.86, 113.70, 113.90, 118.53, 121.92, 127.09, 127.34, 127.70, 127.89, 128.72, 130.49, 131.02, 132.71, 132.84, 134.69, 141.44, 141.83, 143.15, 147.22, 156.90, 159.33 ppm. HRMS (ESI, TOF) m/z calcd for C_27_H_23_BN_3_O, 416.1934; found 416.1933.

#### 2-(3-(pyridin-4-yl)-1H-pyrazol-5-yl) pyridine Boron Complex (P_4_)

Starting with C_4_ (15 mg, 0.7 mmol) and triphenyl borane (16 mg, 0.7 mmol) in toluene, the crude material was collected and purified using column chromatography (silica, ethyl acetate: *n*-hexane 1:1) to afford P_4_ as an off white solid (052 mg, 19%). ^1^H NMR (400 MHz, CDCl_3_) 7.13–7.19 (m, 1H, ArH), 7.23–7.29 (m, 6H, ArH), 7.33–7.39 (m, 5H, ArH), 7.45 (s, 1H, C =CH), 7.68 (t, 1H, *J* = 7.7 Hz, ArH), 7.84 (d, 1H, *J* = 8.1 Hz, ArH), 8.05 (t, 1H, *J* = 7.7 Hz, ArH), 8.20 (d, 1H, *J* = 8.1 Hz, ArH), 8.50 (d, 1H, *J* = 5.9 Hz, ArH), 8.61 (d, 1H, *J* = 4.8 Hz, ArH) ppm. ^13^C NMR (100 MHz, CDCl_3_) 99.77, 119.09, 121.16, 122. 44, 122.68, 127.60, 128.21, 133.30, 136.97, 142.11, 142.36, 143.61, 147.98, 149.51, 153.42, 158.25 ppm. HRMS (ESI, TOF) m/z calcd for C_25_H_20_BN_4_, 387.1781; found 387.1781.

#### 2-(6-Methoxynaphthelene) 5-(2-pyridyl) pyrazolate boron Complex (P_5_)

Starting with C_5_ (20 mg, 0.7 mmol) and triphenyl borane (16 mg, 0.7 mmol) in toluene, the crude material was collected and purified using column chromatography (silica, ethyl acetate: *n*-Hexane 1:1) to afford P_5_ as an off white solid (17 mg, 54%). ^1^H NMR (400 MHz, CDCl_3_) 3.92 (s, 3H, -OCH_3_), 7.11–7.14 (m, 3H, ArH), 7.22–7.29 (m, 7H, ArH), 7.34–7.39 (m, 5H, ArH), 7.75 (d, 1H, *J* = 8.5 Hz, ArH), 7.78 (d, 1H, *J* = 4.8 Hz, ArH), 7.82 (tt, 1H, *J* = 8.1, 0.9 Hz, ArH), 8.01–8.07 (m, 2H, ArH), 8.31 (s, 1H, ArH), 8.51–8.53 (m, 1H, ArH) ppm. ^13^C NMR (100 MHz, CDCl_3_) 53.44, 55.30, 97.40, 105.78, 118.39, 118.82, 121.73, 124.17, 125.16, 126.88, 127.09, 127.75, 127.94, 129.12, 129.62, 129.72, 132.88, 134.09141.54, 141.76, 143.17, 147.56, 157.53, 157.59 ppm. HRMS (ESI, TOF) m/z calcd for C_31_H_25_BN_3_O, 466.2091; found 466.2099.

#### 2-(5-(anthracen-2-yl)-1H-pyrazol-3-yl) pyridine (P_6_)

Starting with C_6_ (20 mg, 0.6 mmol) and triphenyl borane (15 mg, 0.6 mmol) in toluene, the crude material was collected and purified using column chromatography (silica gel, ethyl acetate: *n*-hexane 1:1) to afford P_6_ as a yellow solid. (27 mg, 92%). ^1^H NMR (400 MHz, DMSO-*d*_6_) 7.18–7.31 (m, 10H, ArH), 7.66 (t, 1H, *J* = 6.9 Hz, ArH), 7.69–7.73 (m 2H, ArH), 7.81 (d, 1H, *J* = 8.9 Hz, ArH), 7.85 (d, 1H, *J* = 8.9 Hz, ArH), 7.98 (d, 1H, *J* = 8.0 Hz, ArH), 8.02 (d, 1H, *J* = 8.4 Hz, ArH), 8.23 (d, 1H, *J* = 8.4 Hz, ArH), 8.26 (d, 1H, *J* = 8.0 Hz, ArH), 8.45 (t, 1H, *J* = 7.9 Hz, ArH), 8.90 (d, 1H, *J* = 5.7 Hz, ArH), 8.94 (d, 1H, *J* = 8.3 Hz, ArH), 9.24 (s, 1H, ArH) ppm. ^13^C NMR (100 MHz, DMSO-*d*_6_) 99.04, 119.08. 119.62, 123.51, 124.62, 124.78, 127.02, 127.11, 127.22, 127.32, 127.39, 127.96, 129.03, 129.43, 130.24, 130.47, 131.41, 132.36, 132.88, 142.59, 144.24, 144.46, 146.41 ppm. HRMS (ESI, TOF) m/z calcd for C_34_H_25_BN_3_, 485.2178; found 486.21337.

## Supplementary Information

Supplementary Information 1.

Supplementary Information 2.

Supplementary Information 3.

Supplementary Information 4.

Supplementary Information 5.

Supplementary Information 6.

Supplementary Information 7.

Supplementary Information 8.

Supplementary Information 9.

Supplementary Information 10.

## Data Availability

All data generated or analysed during this study are included in this published article and its supplementary information file. The data is also available through a request from the corresponding author.
