# Supplementary material for: Synthesis and photophysical investigations of pyridine-pyrazolate bound boron(III) diaryl complexes
Source: Sci Rep. 2022 Oct 1;12:16482. doi: 10.1038/s41598-022-20796-2 (PMC9526719; doi:10.1038/s41598-022-20796-2)
Supplement: Supplementary file 3 — Supplementary Information 3. [file 41598_2022_20796_MOESM3_ESM.docx]

Synthesis and Photophysical Investigations of Pyridine-Pyrazolate Bound Boron(III) Diaryl Complexes

Rashid Javaid, ^A^* Aziz Ul Rehman, ^B,C^ Manan Ahmed, ^D^ Mohammad Hashemi Karouei, ^A^ Nima Sayyadi, ^A,B^

1. Department of Molecular Sciences, Macquarie University, New South Wales, 2109, Australia.
2. ARC Centre of Excellence for Nanoscale Bio photonics (CNBP), Macquarie University, New South Wales, 2109, Australia.
3. Agri. & Biophotonics Division, National Institute of Lasers and Optronics College, Pakistan Institute of Engineering and Applied Sciences (PIEAS), 45650 Islamabad, Pakistan
4. School of Chemistry, University of New South Wales, Sydney, New South Wales 2052, Australia

* Corresponding author’s email: rashidjavaid143@gmail.com

Table of Contents

[Appendix 2](#_Toc58786100)

[Figures 29](#_Toc58786101)

[References 30](#_Toc58786102)

# Appendix

**Characterization of the compounds:**

All synthesized compounds were characterized using nuclear magnetic resonance (400 MHz for proton, and 100 MHz for carbon) (NMR, ^1^H, ^13^C). CDCl_3_ and DMSO were used as solvents, CDCl_3_ was deacidified by passing it through and their peaks were referenced according to literature.^1^ The purity was further confirmed by HRMS analysis. High-resolution mass spectra were recorded with a mass spectrometer (Agilent 6538 Q-TOF with dual ESI source).


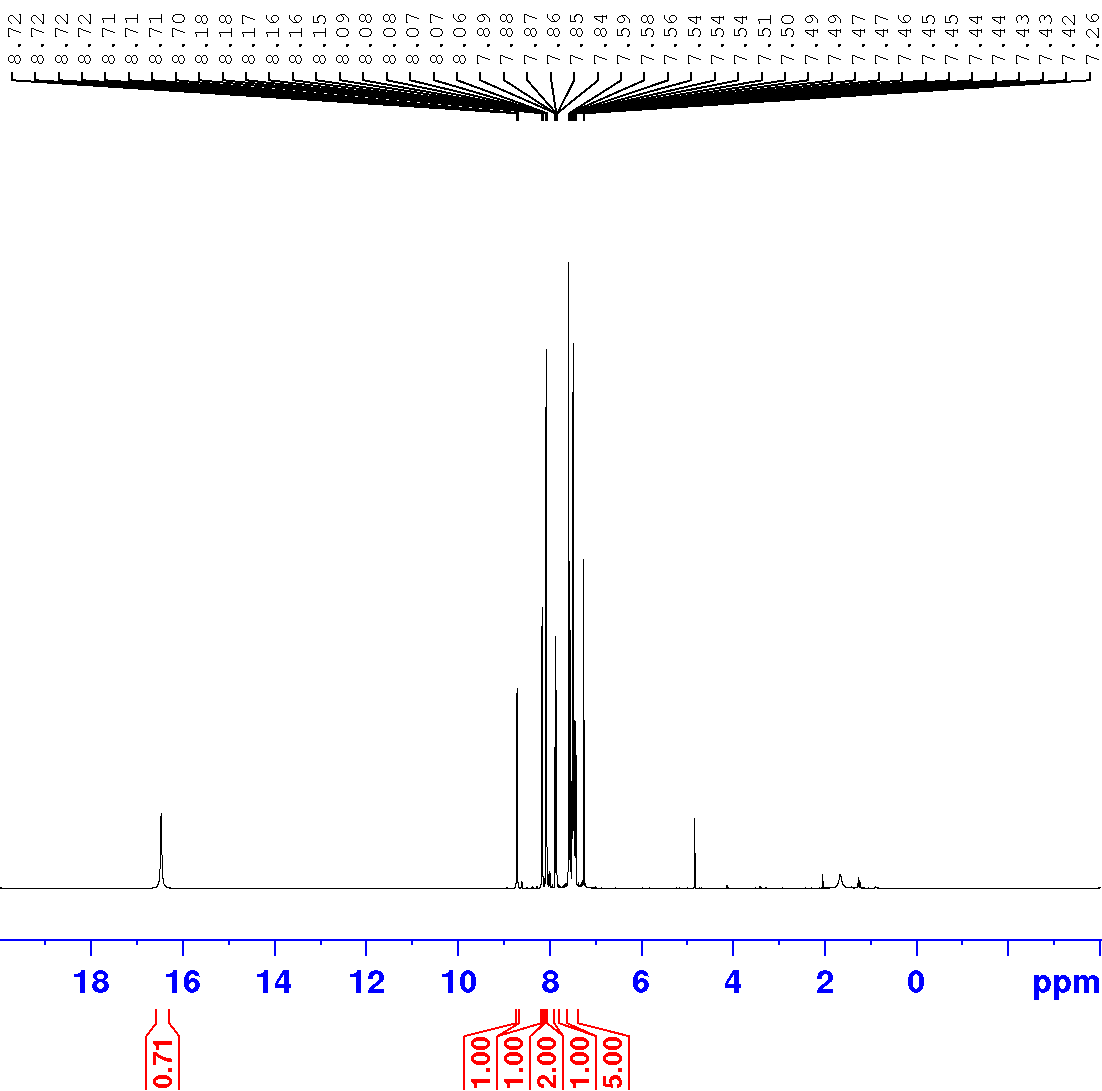


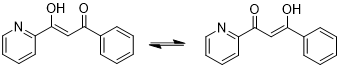


-OH proton of enol

**Figure A2.1:** ^1^H NMR spectrum of B_1_ in deacidified CDCl_3_. Distinct enolic proton confirmed the product formation.


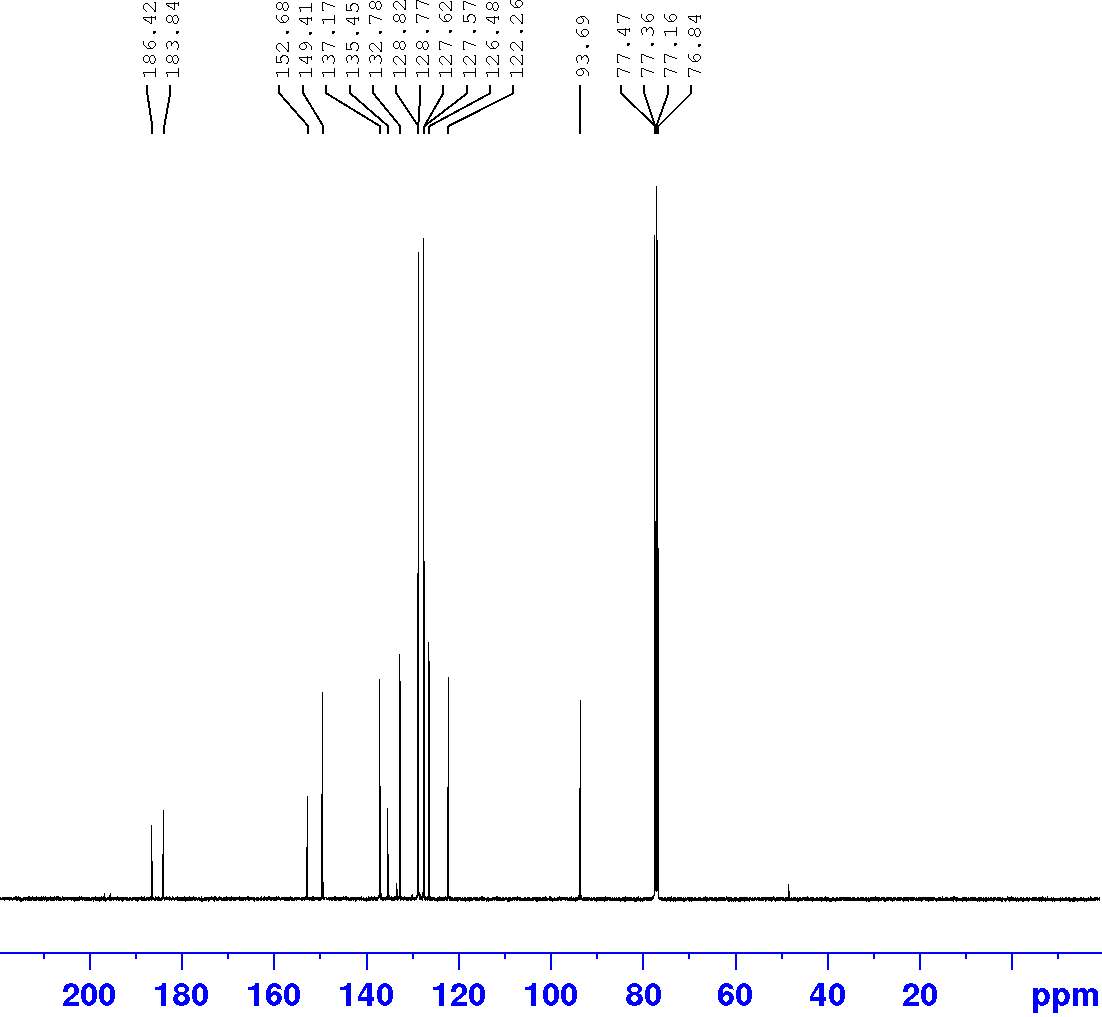


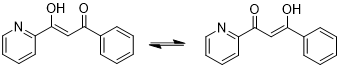


**Figure A2.2:** ^13^**C** NMR spectrum of B_1_ in deacidified CDCl_3_.

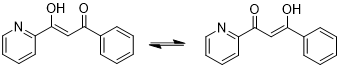


**Figure A2.3:** HRMS spectrum of B_1_ confirming the product due to appearance of peak at 226.0868 in agreement with the calculated value.


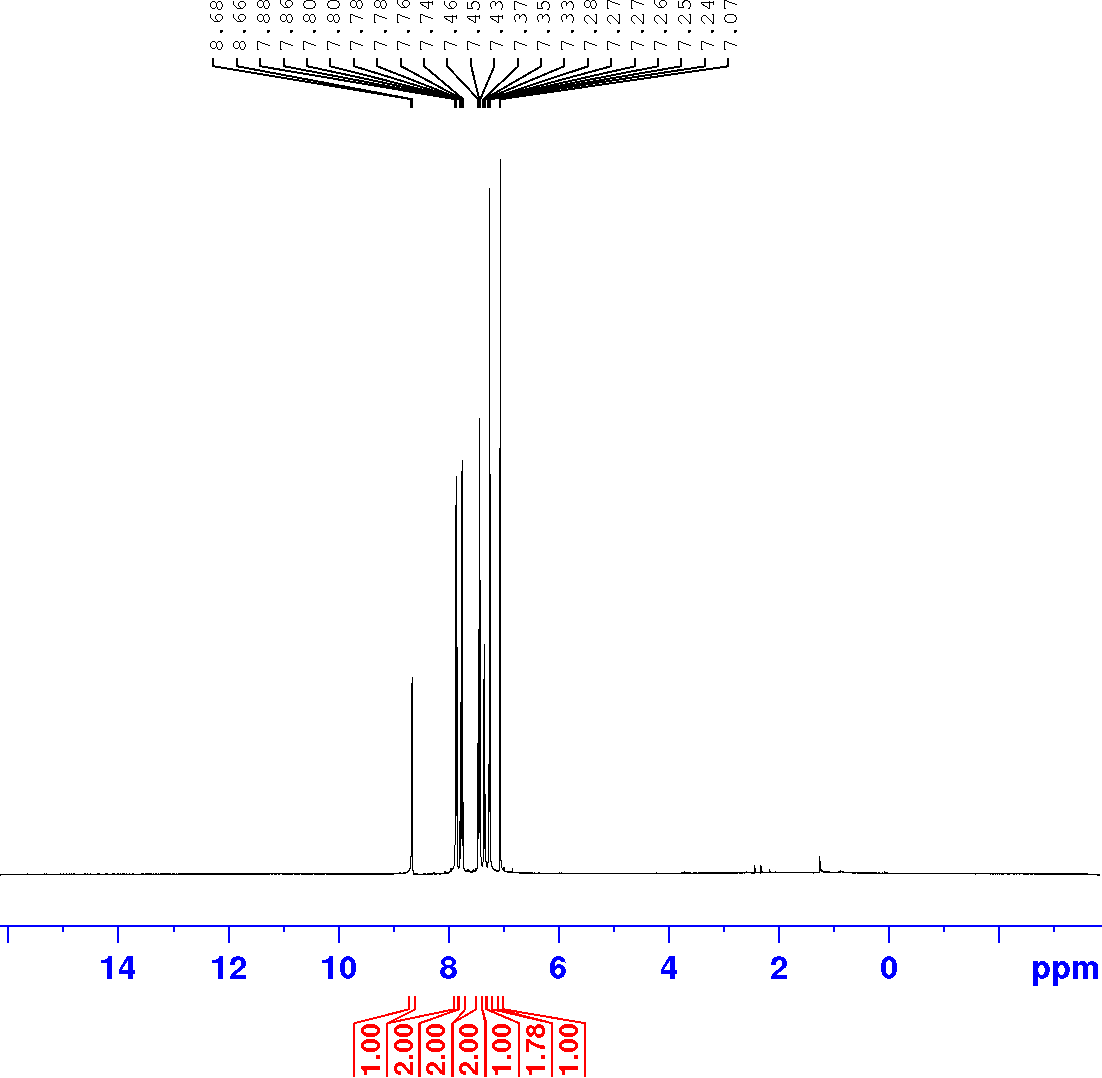


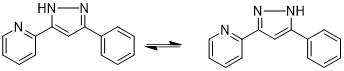


**Figure A2.4:** ^1^H NMR spectrum of C_1_ in deacidified CDCl_3_. The product formation was confirmed by the disappearance of enolic proton and upfield shifting of protons due to electron donating nature of nitrogen compared to oxygen.


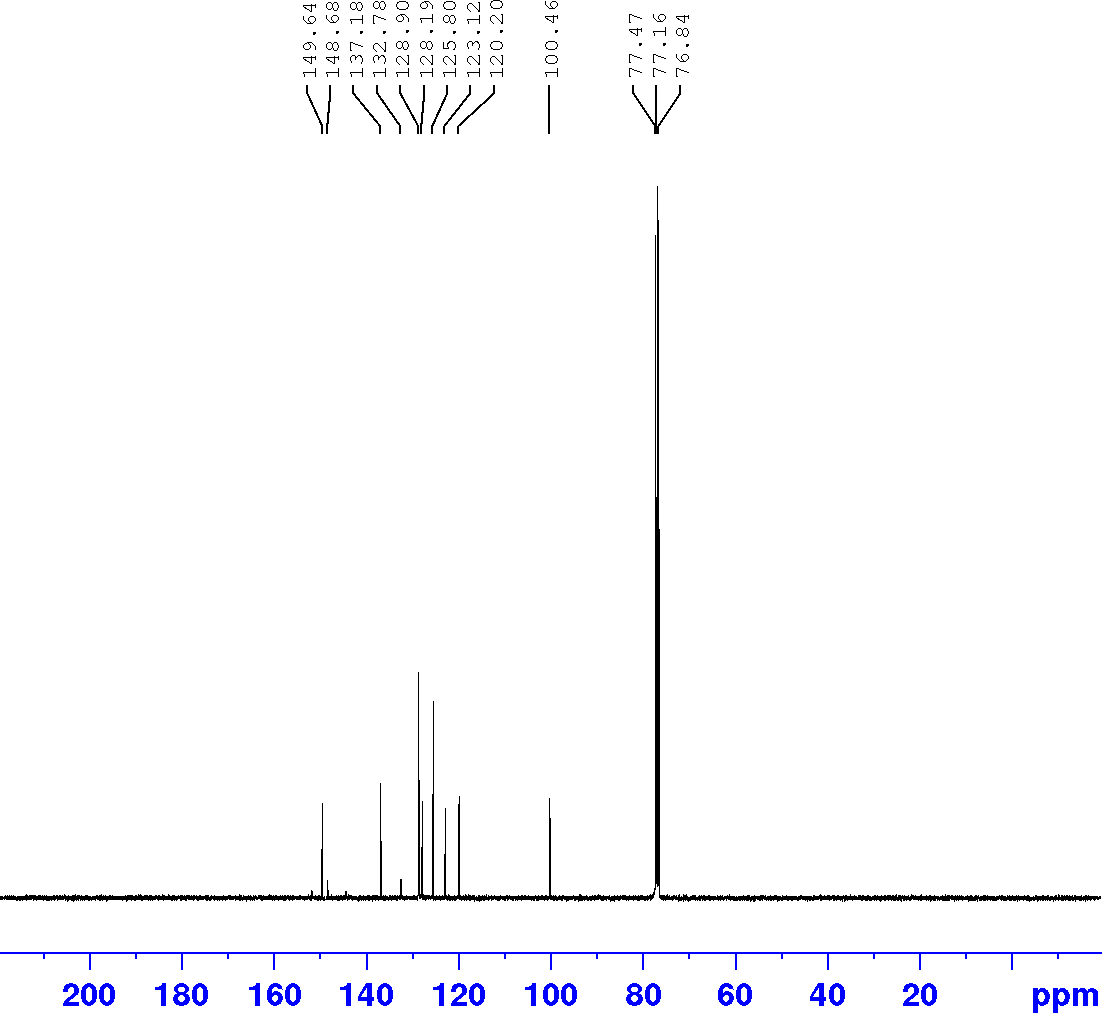


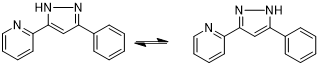


**Figure A2.5:** ^13^**C** NMR spectrum of C_1_ in deacidified CDCl_3_.

]


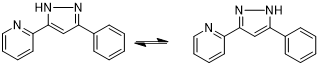


**Figure A2.6:** HRMS spectrum of C_1._ Product formation was confirmed by the peak at 222.10312 which agrees with the calculated value.


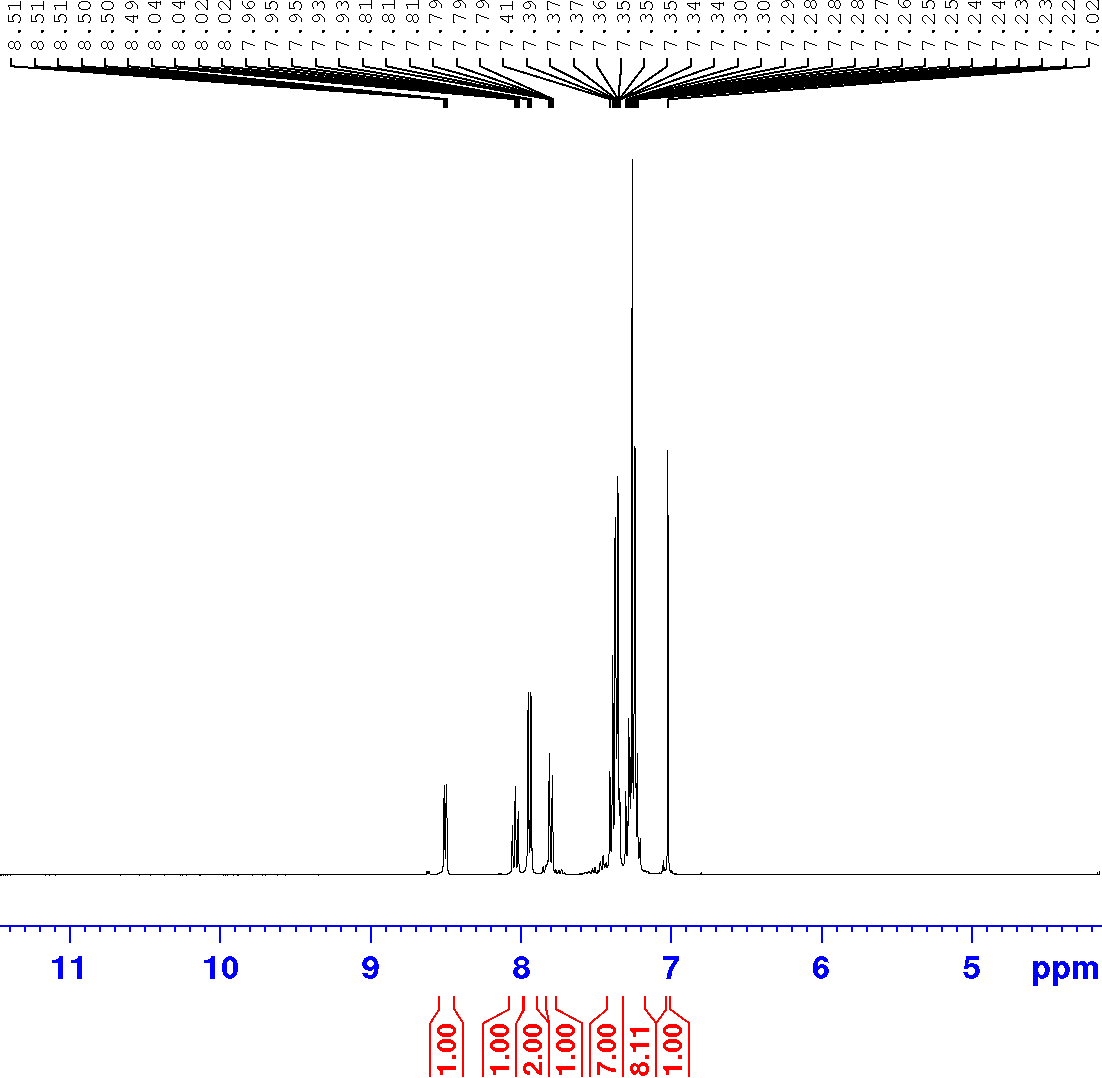

**Figure A2.7:** ^1^H NMR spectrum of P_1_ in deacidified CDCl_3._ The product formation was confirmed comparing the NMR of the ligand which clearly shows distinct protons and their shifting upfield.


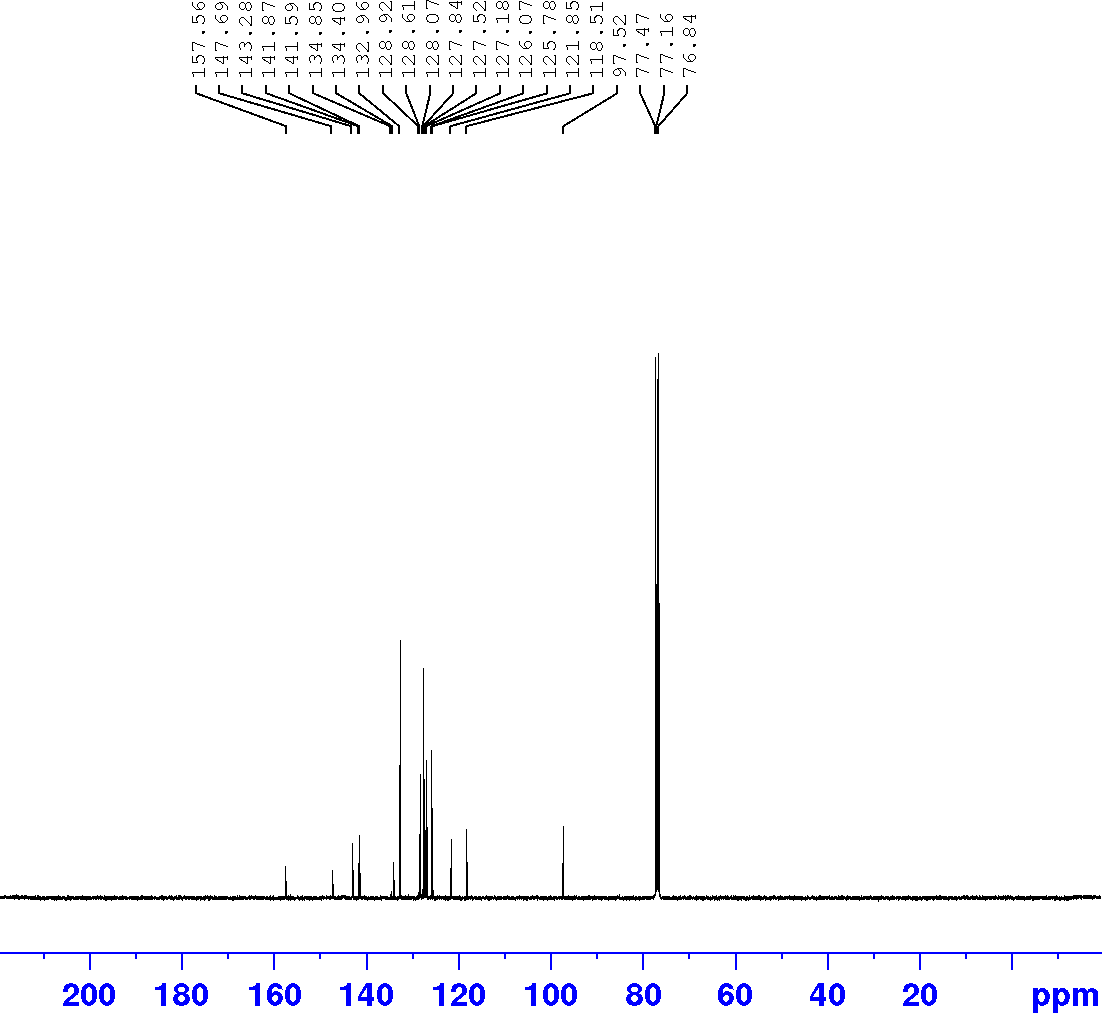

**Figure A2.8:** ^13^**C** NMR spectrum of P_1_ in deacidified CDCl_3_

**Figure A2.9:** HRMS spectrum of P_1._ The product formation was confirmed by the peak at 386.18240 which agrees the calculated value.


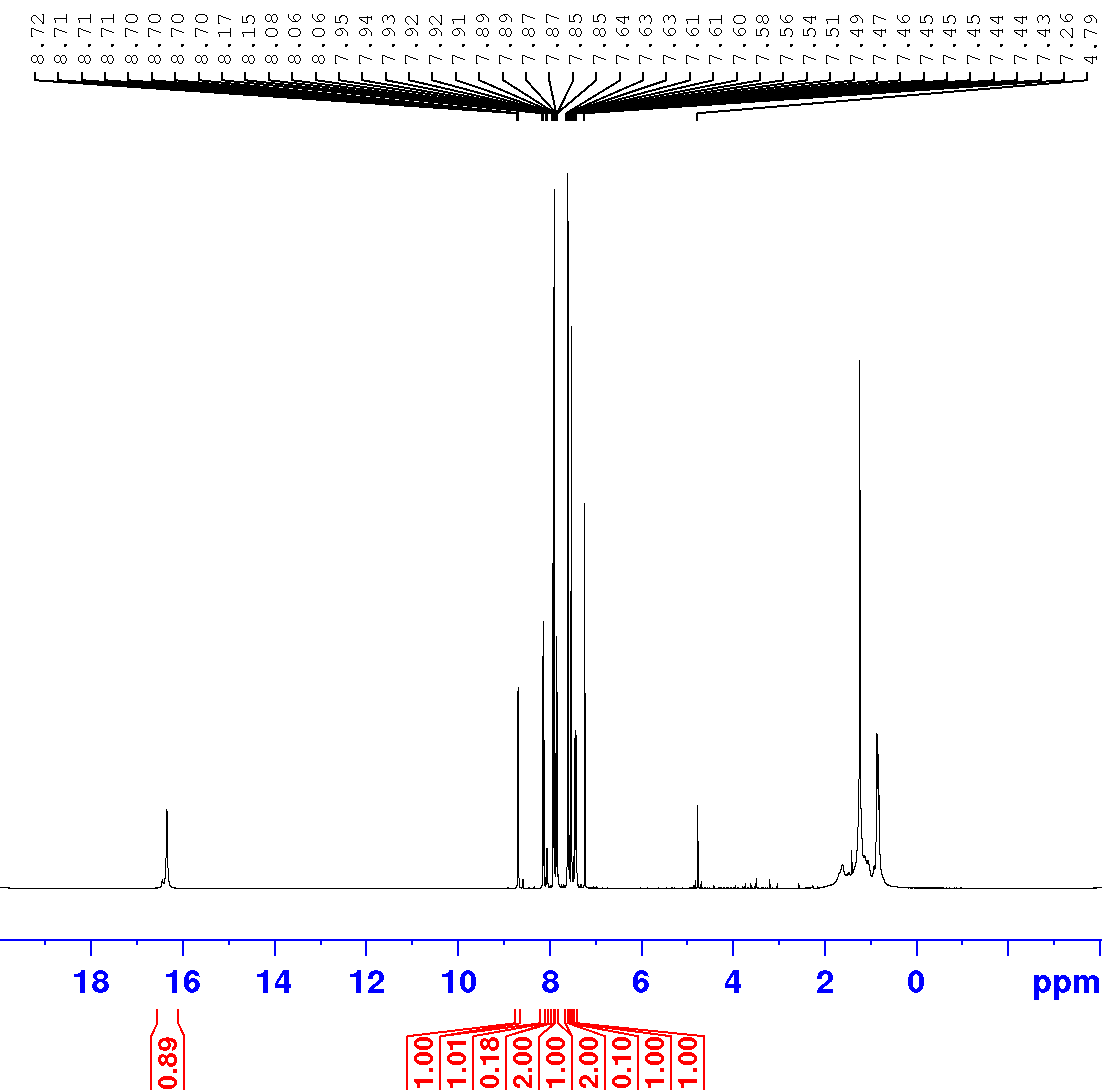

-OH proton of enol

**Figure A2.10:** ^1^H NMR spectrum of B_2_ in deacidified CDCl_3_. Distinct enolic proton confirmed the product formation.


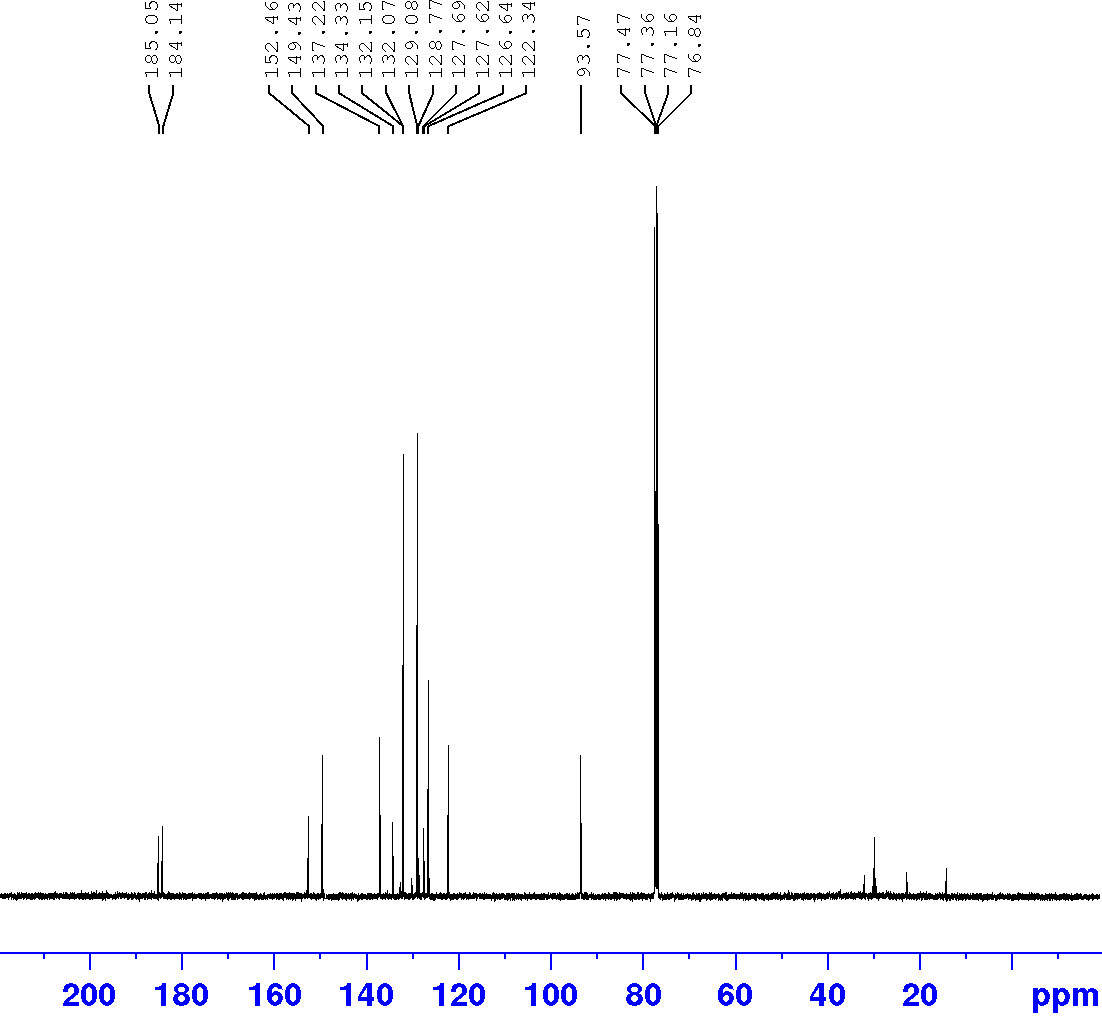

**Figure A2.11:** ^13^**C** NMR spectrum of B_2_ in deacidified CDCl_3_.

**Figure A2.12:** HRMS spectrum of B_2._ confirmed by the peak at 303.99662 which agrees the calculated values.


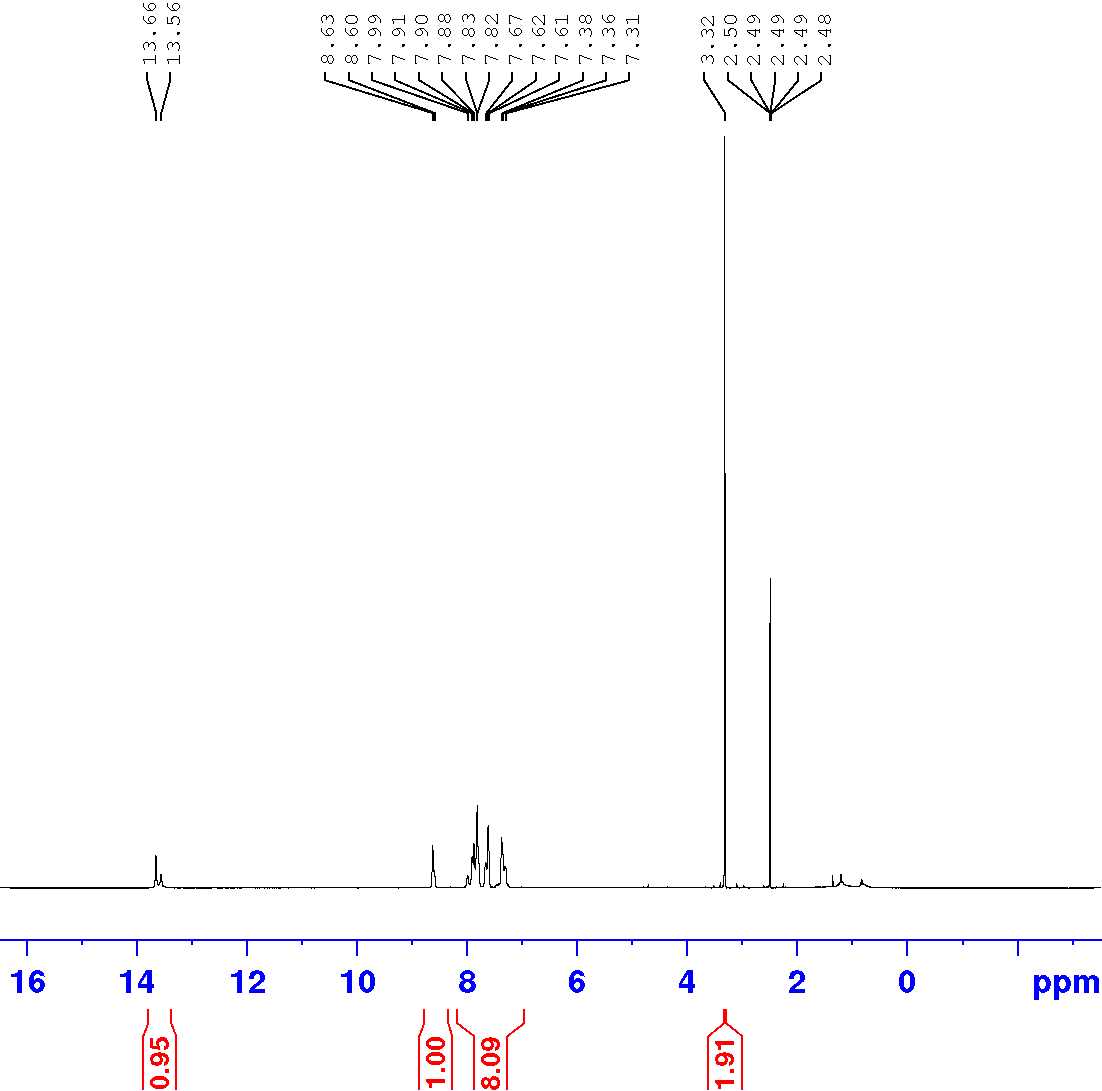


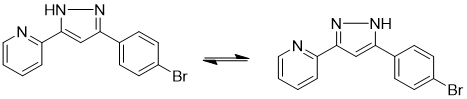


-NH proton of pyrazole

**Figure A2.13:** ^1^H NMR spectrum of C_2_ in deacidified CDCl_3_. The product formation was confirmed by the disappearance of enolic proton, the appearance of -NH proton and upfield shifting of peaks due to replacement of electronegative oxygen with nitrogen.


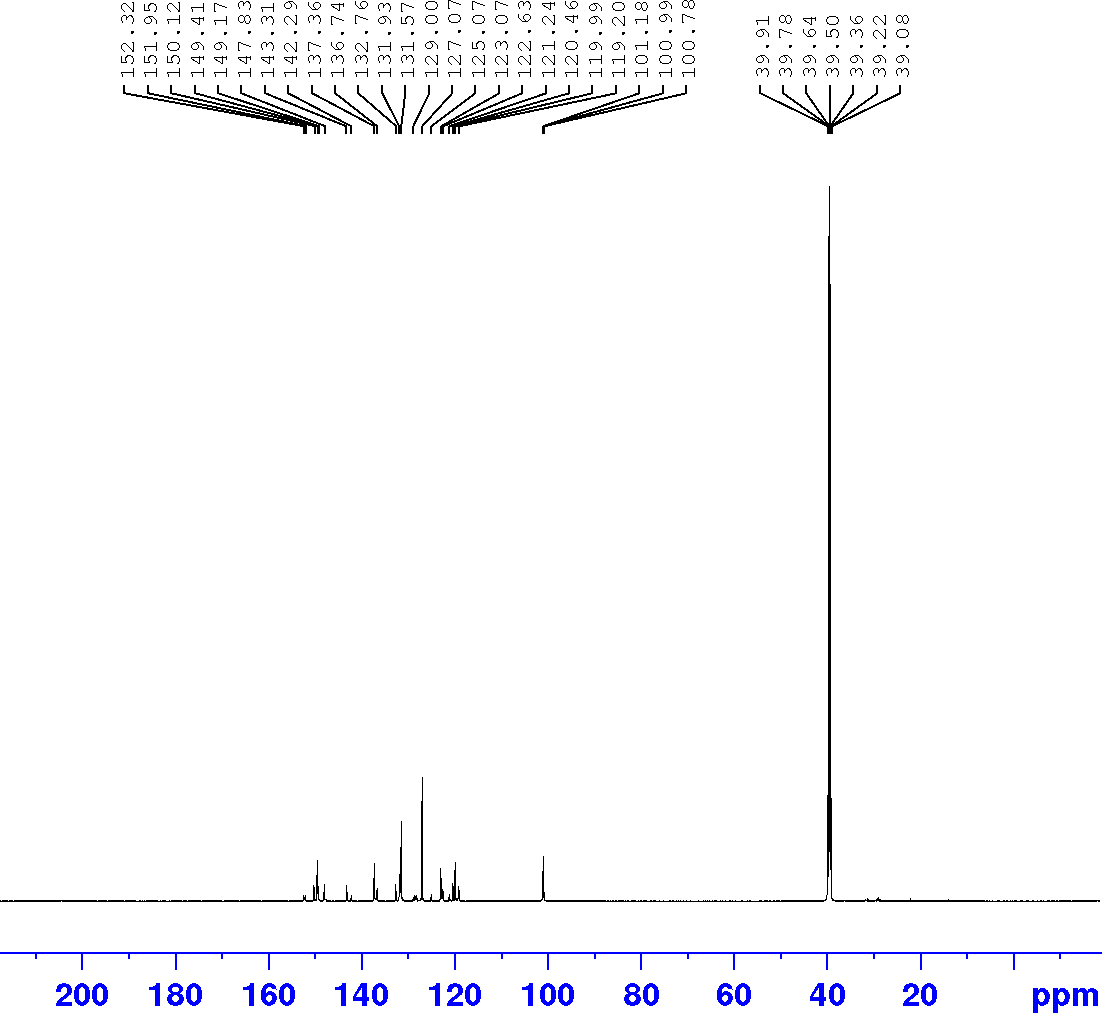


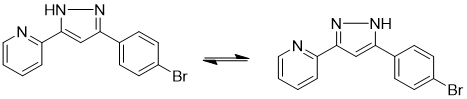


**Figure A2.14:** ^13^**C** NMR spectrum of C_2_ in deacidified CDCl_3_.

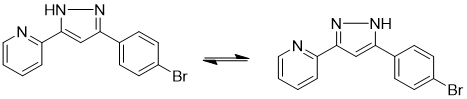


**Figure A2.15:** HRMS spectrum of C_2_ confirmed by the appearance of peak at 300.01290 which agrees the calculated values.


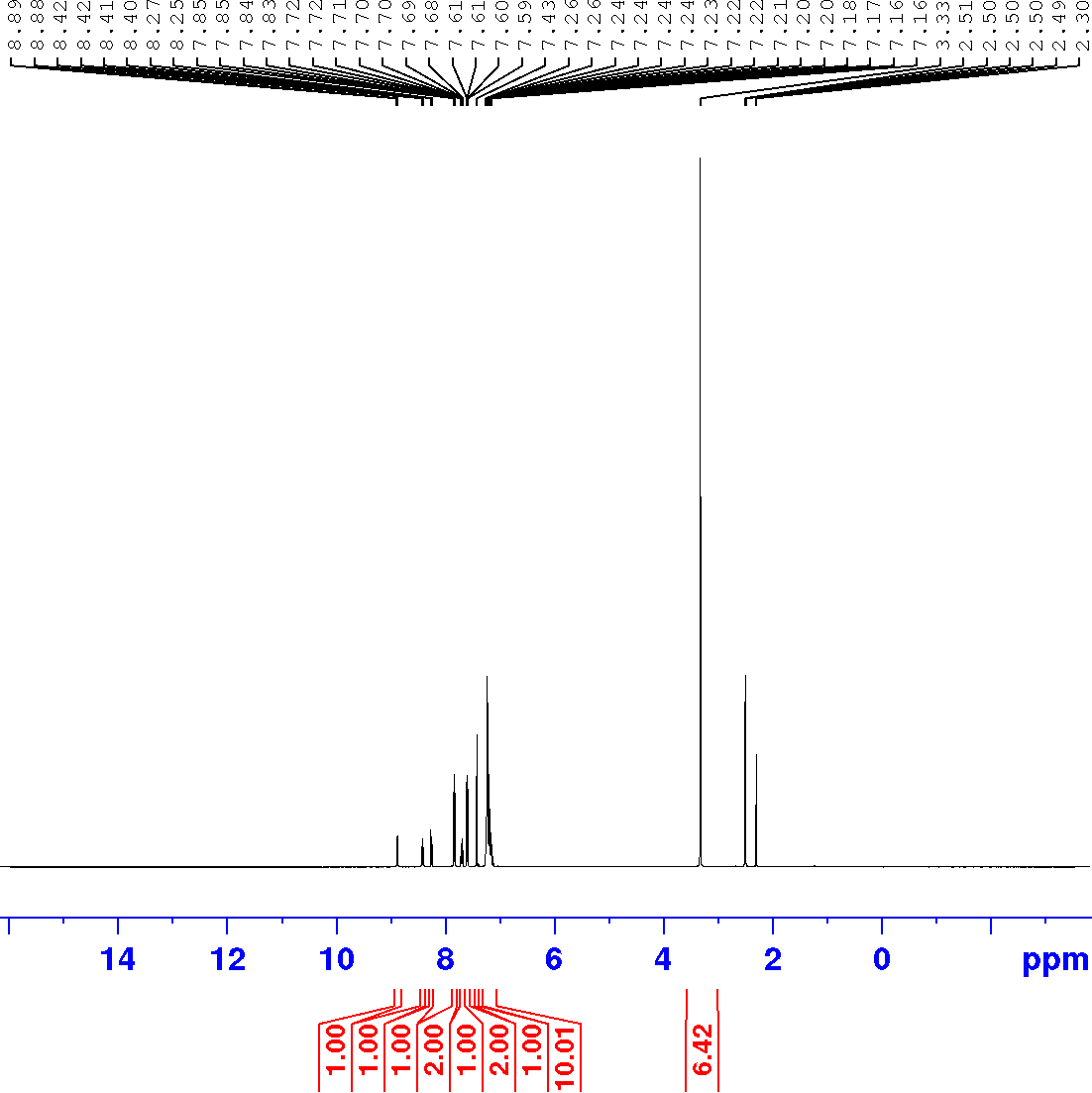

**Figure A2.16:** ^1^H NMR spectrum of P_2_ in DMSO-*d*_6_ . The product formation was confirmed by the disappearance of -NH proton of the ligand and further upfield shifting of protons due to formation of electron rich molecule.


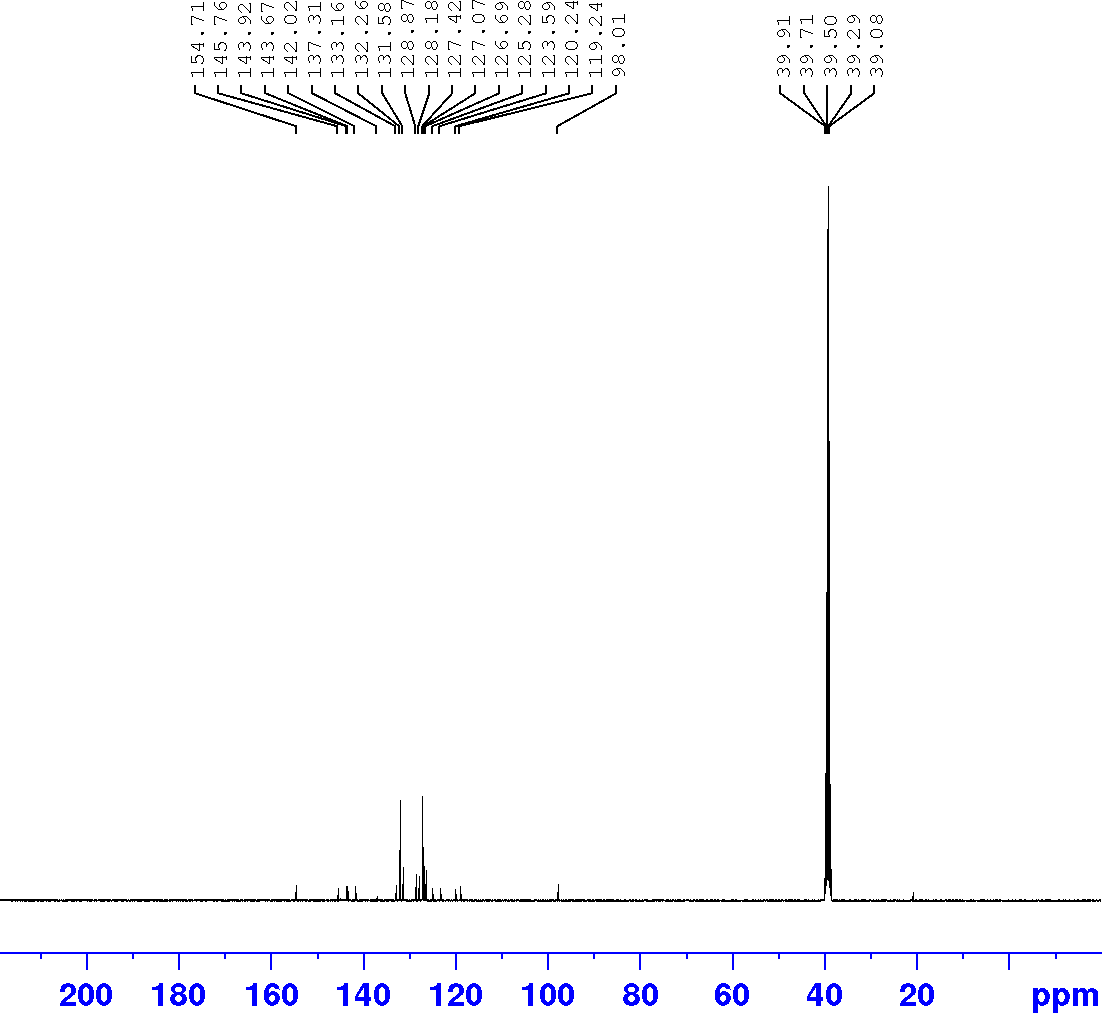

**Figure A2.17:** ^13^**C** NMR spectrum of P_2_. in DMSO-*d*_6_

**Figure A2.18:** HRMS spectrum of P_2._ The product formation was confirmed by the peak at 464.0941 which agrees the calculated value.


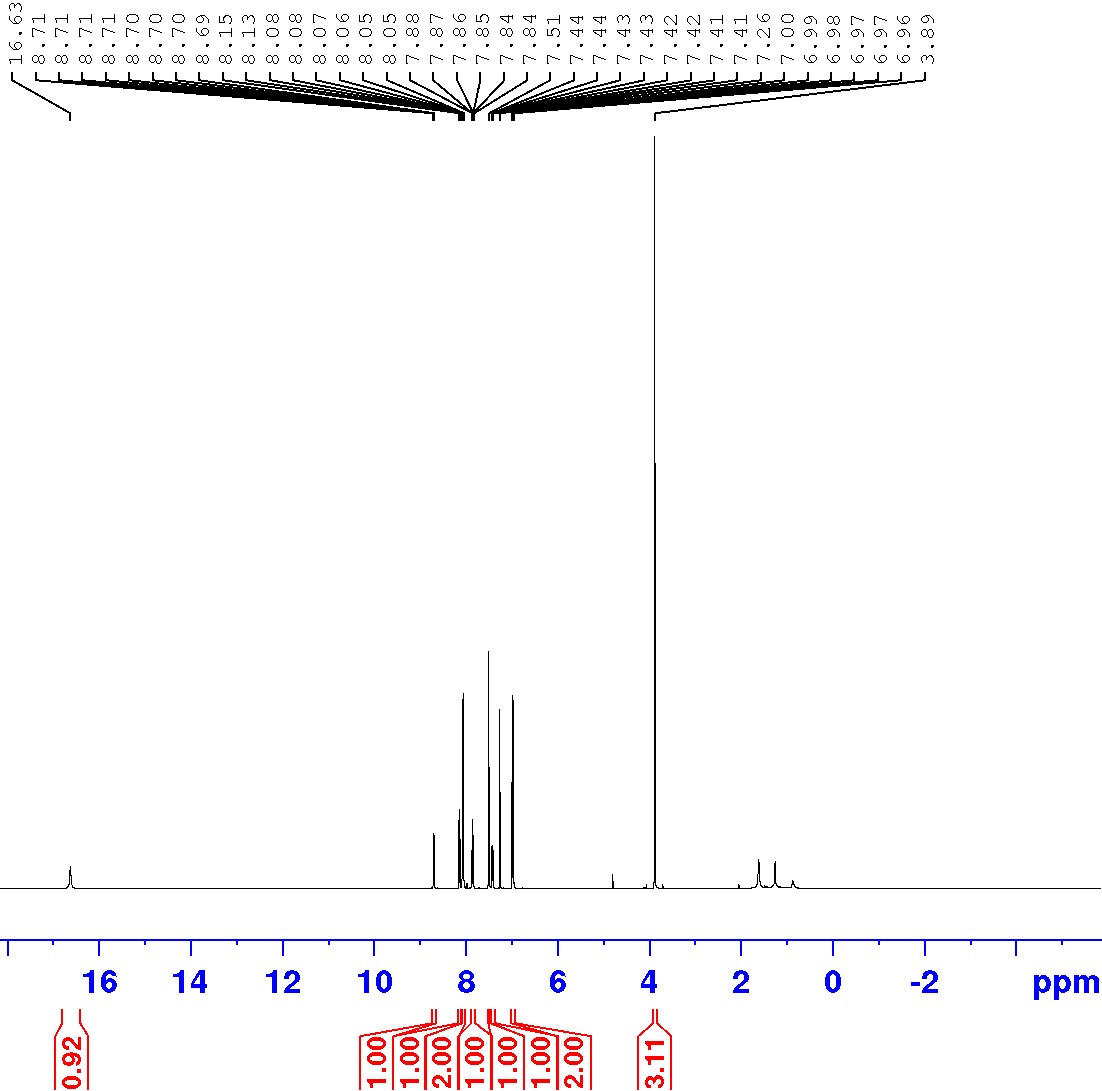

-OCH_3_

-OH proton of enol

**Figure A2.19:** ^1^H NMR spectrum of B_3_ in deacidified CDCl_3_. The product formation was confirmed by the appearance of characteristic enolic proton.


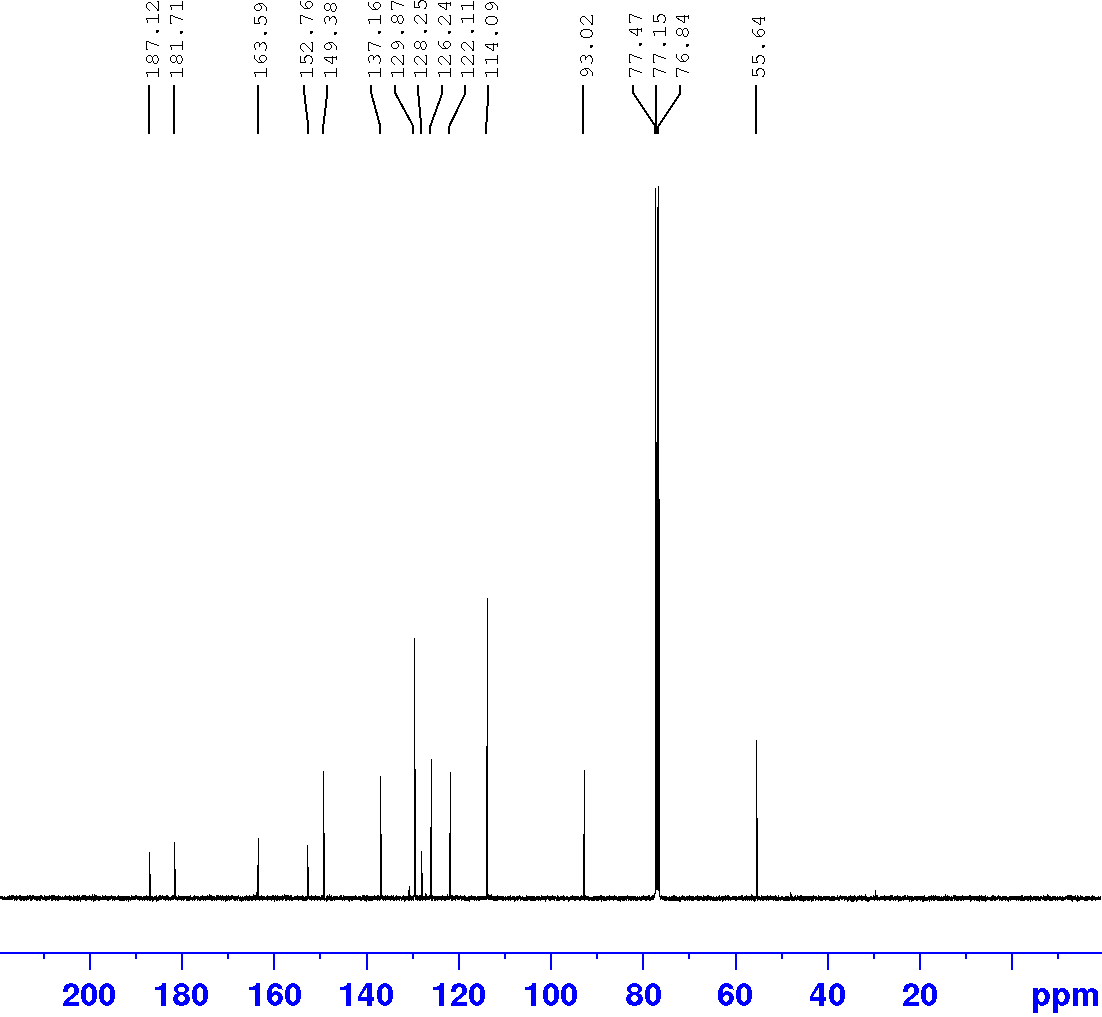

**Figure A2.20:** ^13^**C** NMR spectrum of B_3_ in deacidified CDCl_3_.

**Figure A2.21:** HRMS spectrum of B_3._ The product formation was confirmed by the peak at 256.09653 which agrees the calculated value.


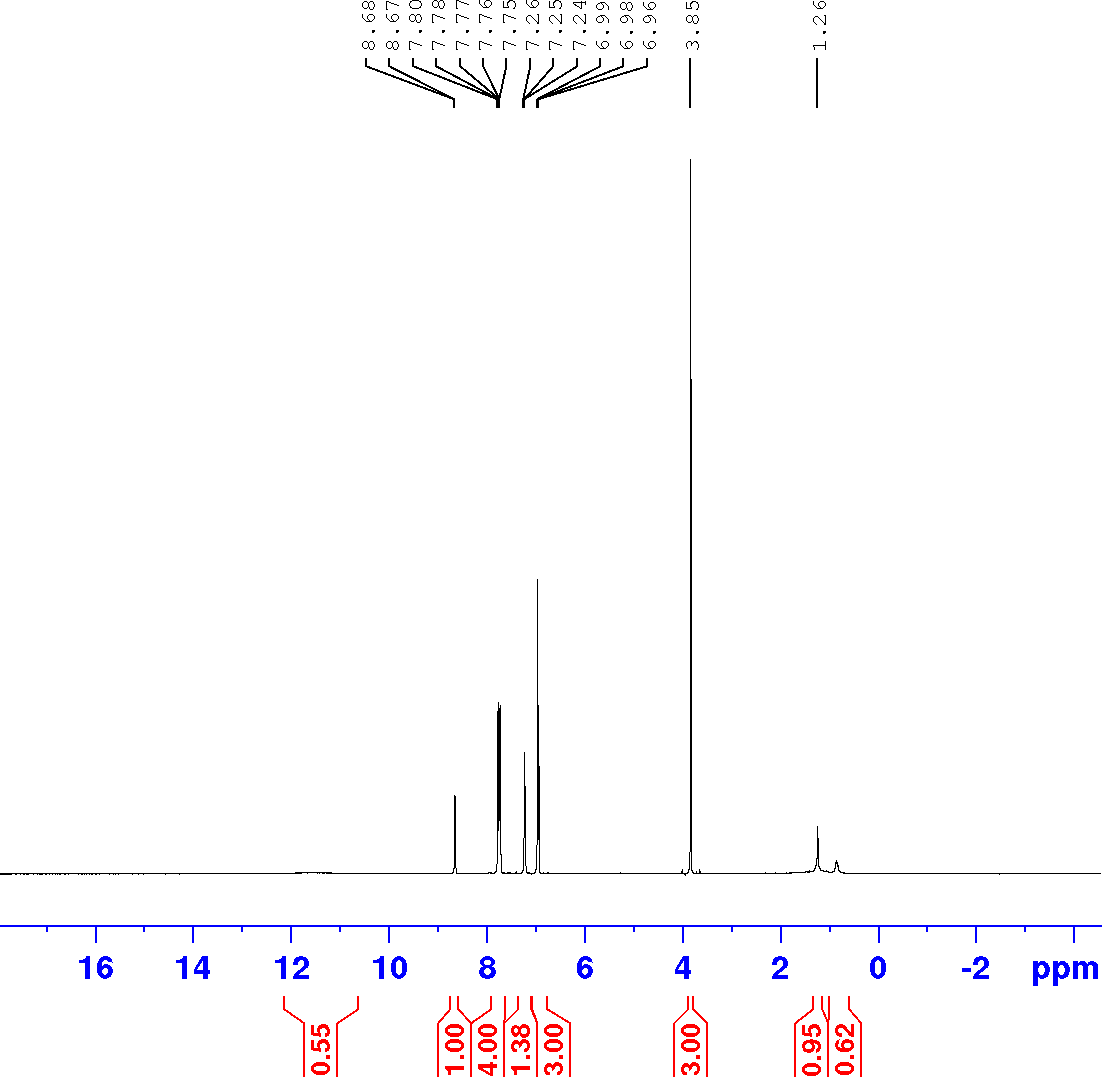

-OCH_3_

-NH proton of pyrazole

**Figure A2.22:** ^1^H NMR spectrum of C_3_ in deacidified CDCl_3_. Formation of the ligand was confirmed by the disappearance of enolic proton, appearance of -NH proton and the upfield shifting of protons due to replacement of electronegative oxygen with nitrogen.


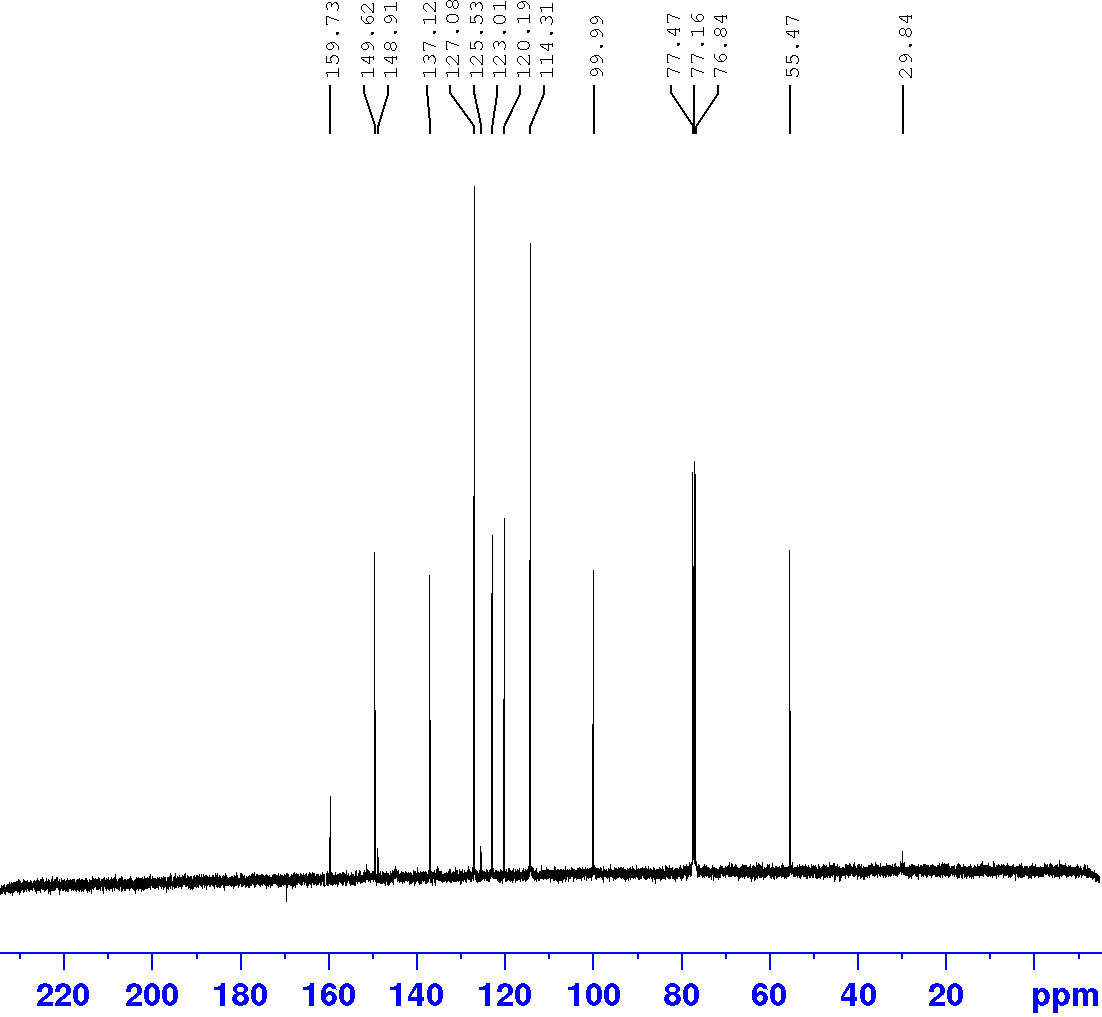

**Figure A2.23:** ^13^**C** NMR spectrum of C_3_ in deacidified CDCl_3_.

**Figure A2.24:** HRMS spectrum of C_3._ Formation of the product was confirmed by the peak at 252.11289 which agrees the calculated value.


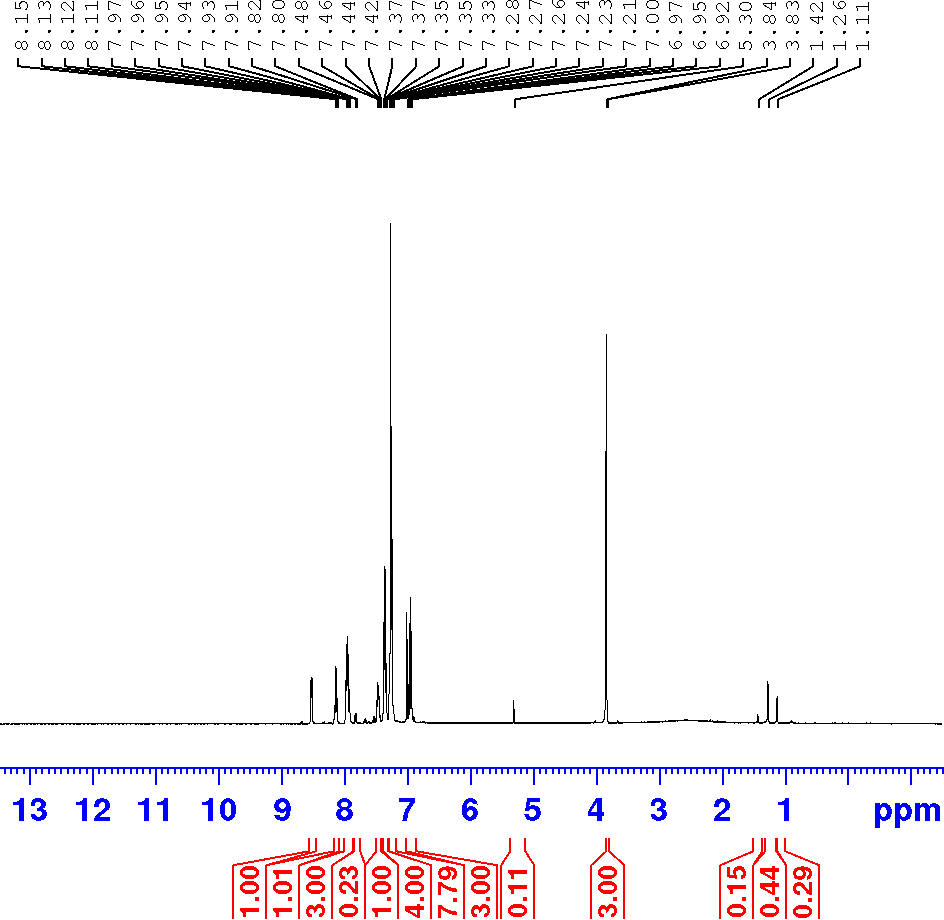

**Figure A2.25:** ^1^H NMR spectrum of P_3_ in deacidified CDCl_3_. Formation of the product was confirmed by the disappearance of -NH proton and further upfield shifting of the peaks compared to ligand due to formation of electron rich molecule.


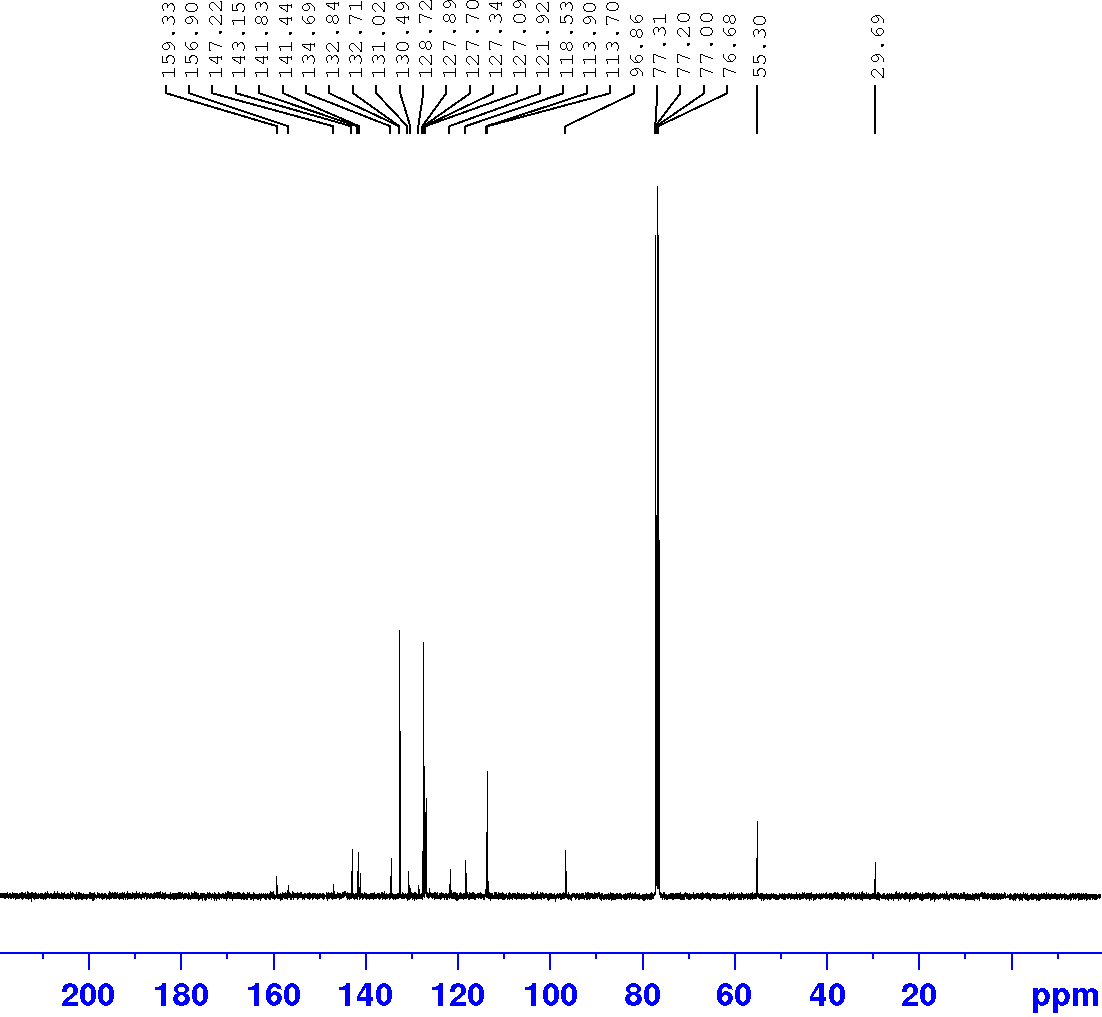

**Figure A2.26:** ^13^C NMR spectrum of P_3_ in deacidified CDCl_3_.

**Figure A2.27:** HRMS spectrum of P3. Formation of the product was confirmed by the peak at 416.1933 which agrees the calculated value.


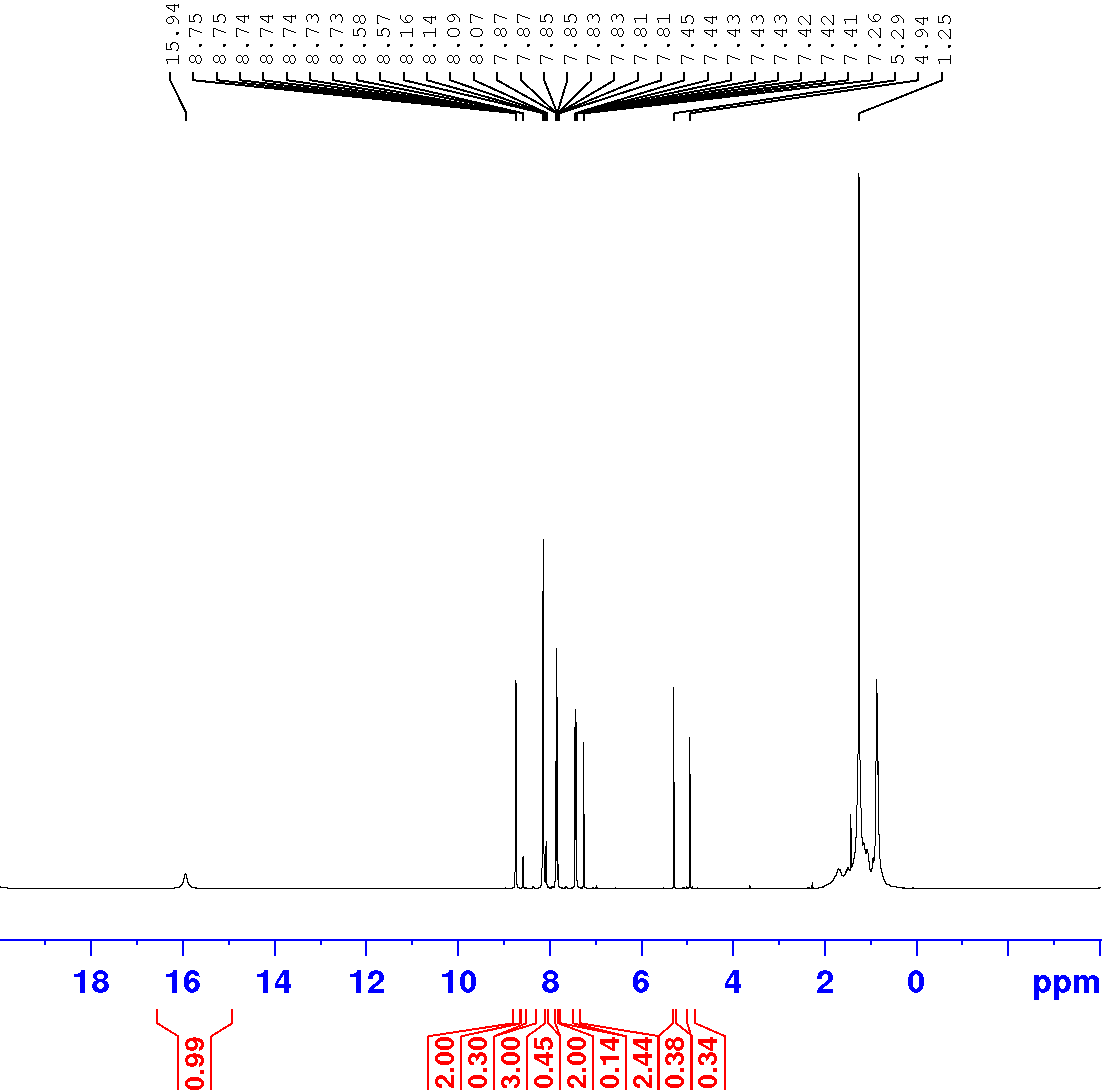


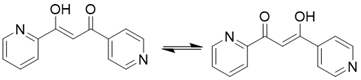


-OH proton of enol

**Figure A2.37:** ^1^H NMR spectrum of B_4_ in deacidified CDCl_3._ The product formation was confirmed by the appearance of distinct enolic proton.


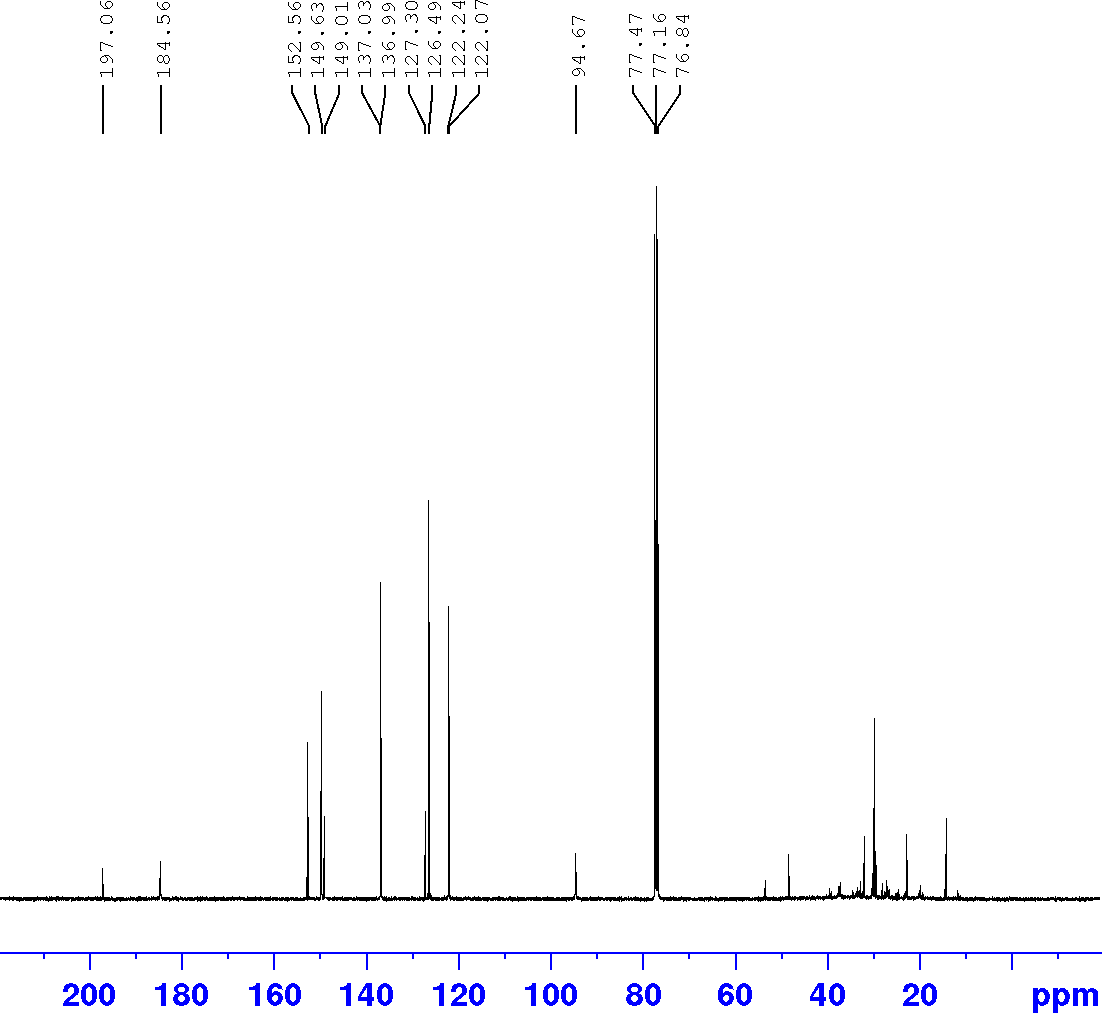


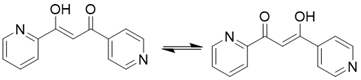


**Figure A2.38:** ^13^C NMR spectrum of B_4_ in deacidified CDCl_3_.

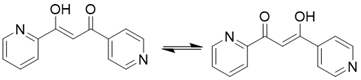


**Figure A2.39:** HRMS spectrum of B_4_. Formation of the product was confirmed by the peak at 227.08128 which agrees the calculated value.


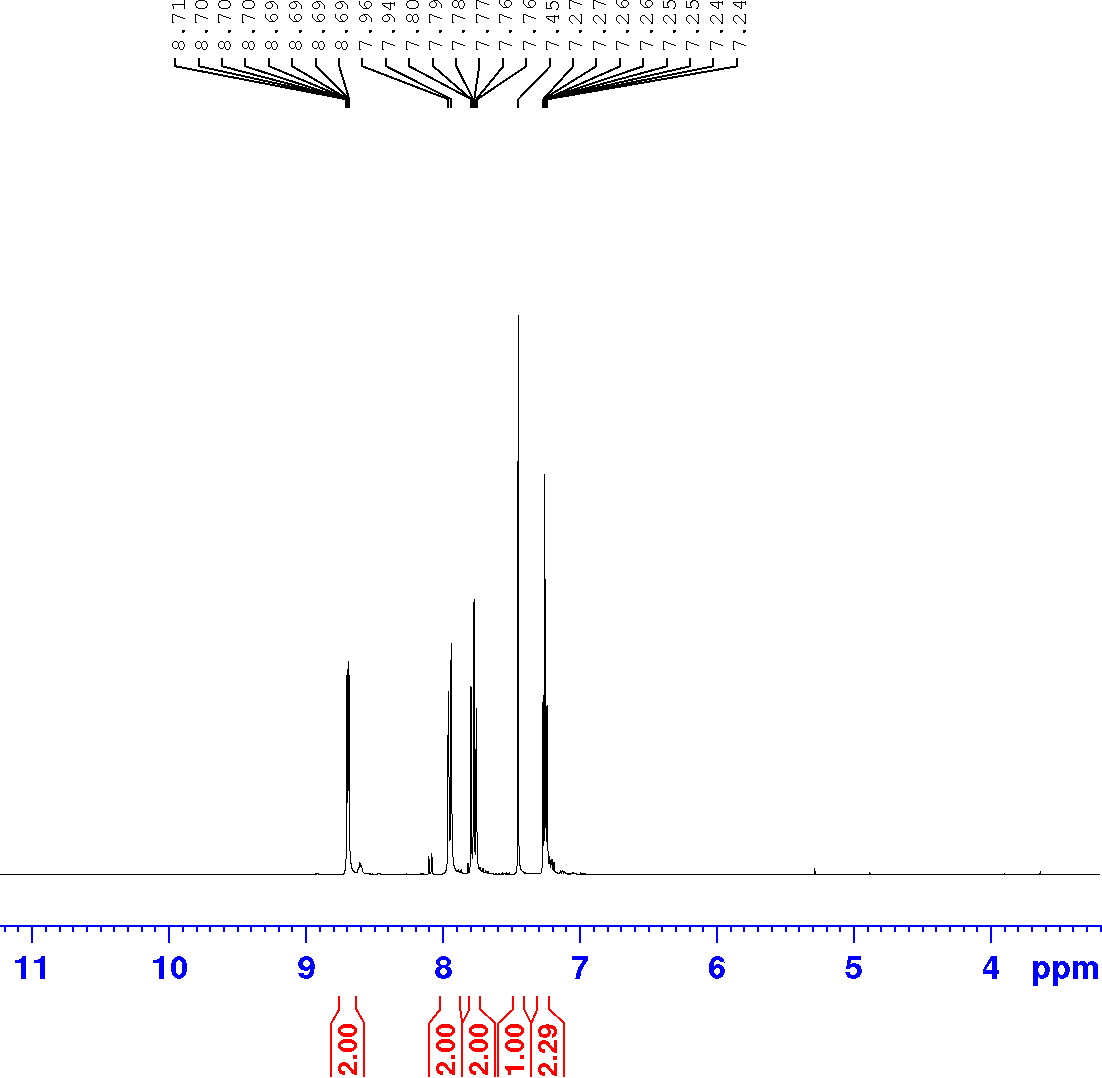


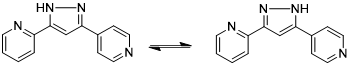


**Figure A2.40:** ^1^H NMR spectrum of C_4_ in deacidified CDCl_3_. Formation of the product was confirmed by the disappearance of the enolic proton and shifting of peaks upfield due to replacement of oxygen with nitrogen.


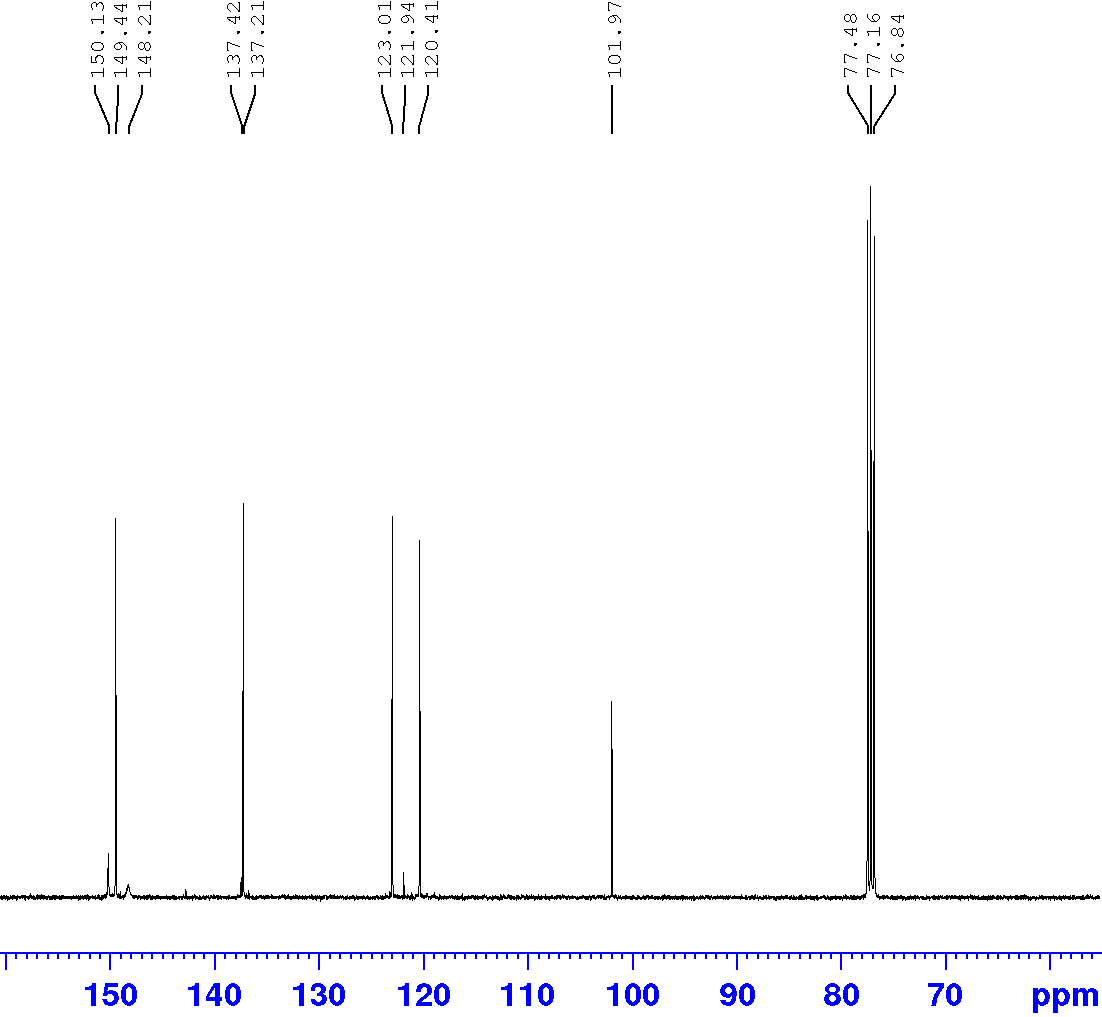


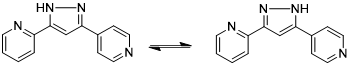


**Figure A2.41:** ^13^C NMR spectrum of C_4_ in deacidified CDCl_3_.

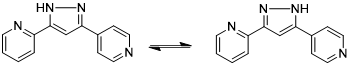


**Figure A2.42:** HRMS spectrum of C_4._ Formation of the product was confirmed by the peak at 223.09772 which agrees the calculated value.


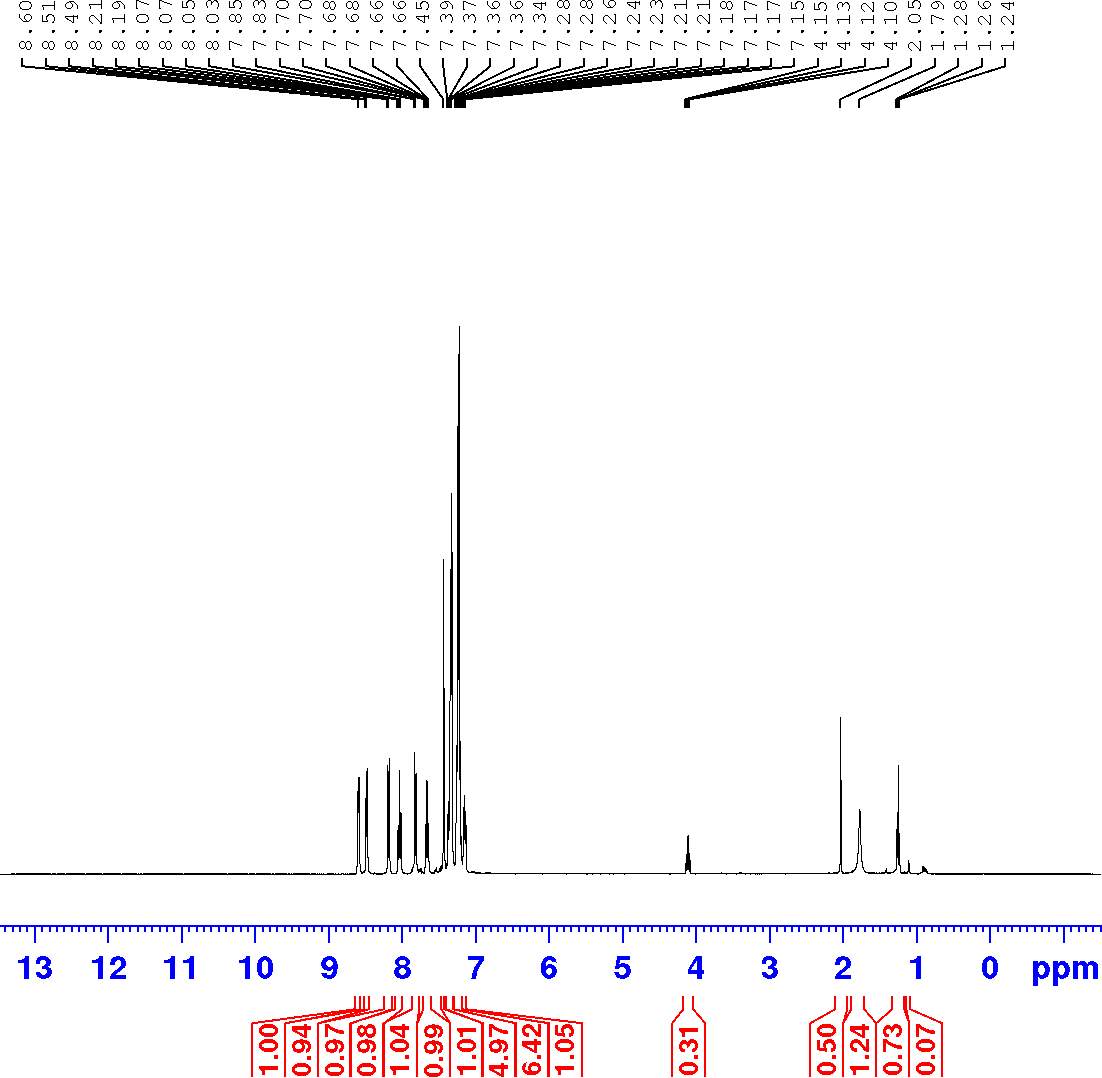


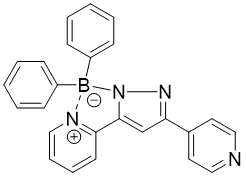


**Figure A2.43:** ^1^H NMR spectrum of P_4_ in deacidified CDCl_3_. The product formation was confirmed by the shifting of protons upfield due to formation of electron rich molecule.


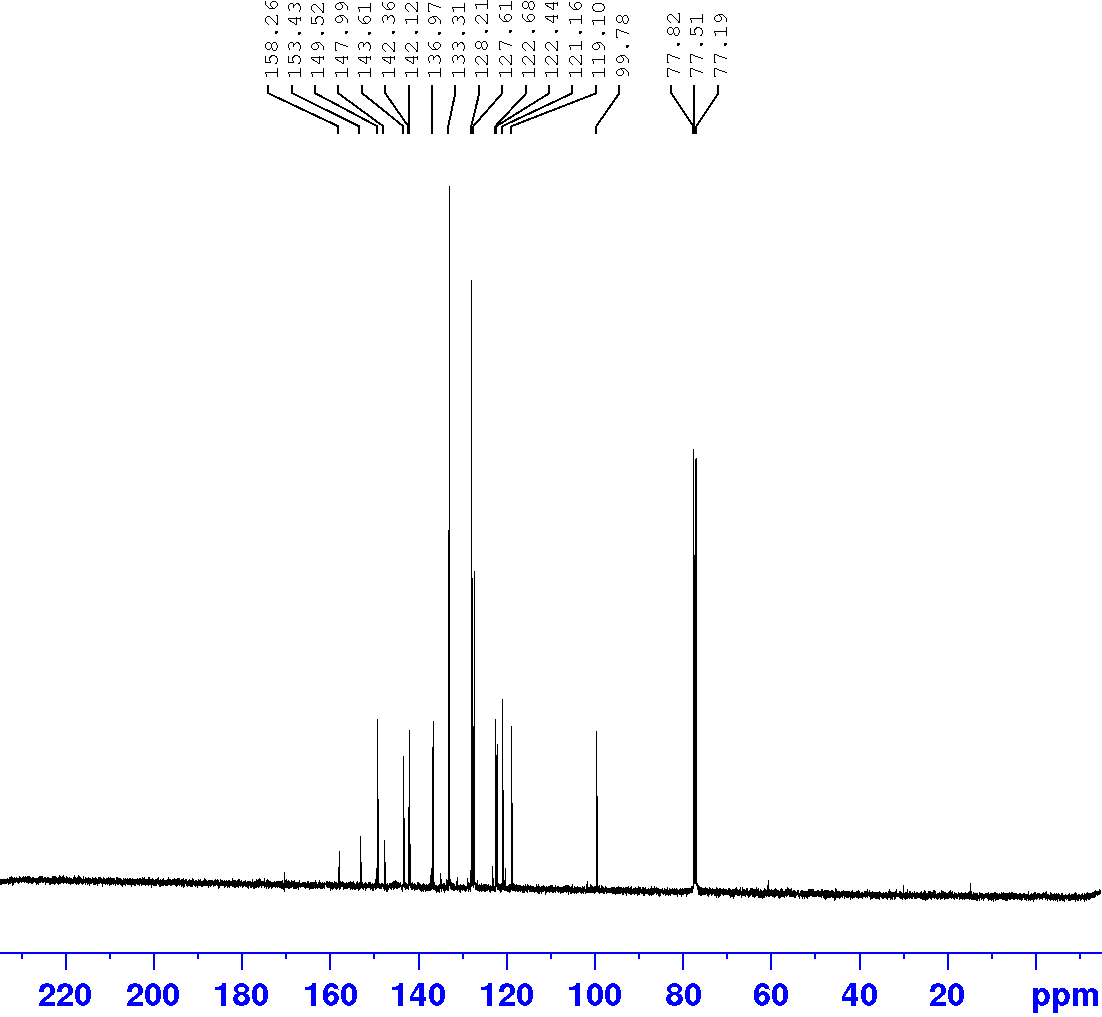


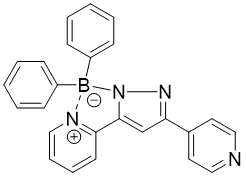


**Figure A2.44:** ^13^C NMR spectrum of P_4_ in deacidified CDCl_3_.

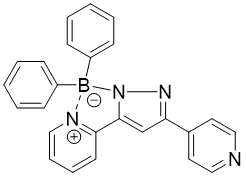


**Figure A2.45:** HRMS spectrum of P_4._ Formation of the product was confirmed by the peak at 387.1781 which agrees the calculated value


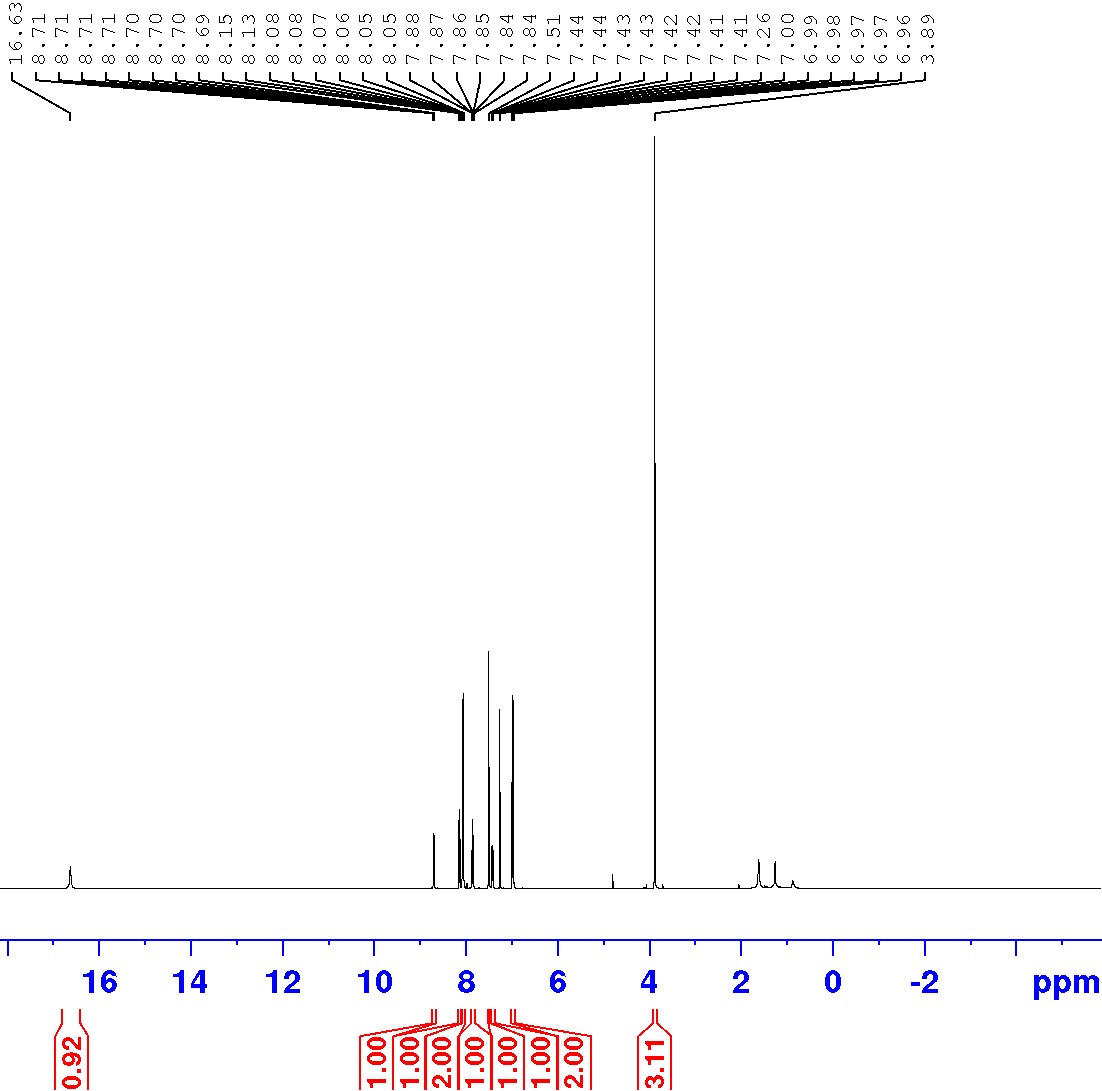


-OCH_3_ proton


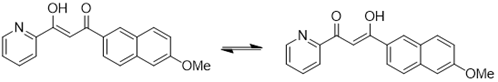


-OH proton of enol

**Figure A2.28:** ^1^H NMR spectrum of B_5_ in deacified CDCl_3_. Formation of the product was confirmed by the characteristic enolic proton


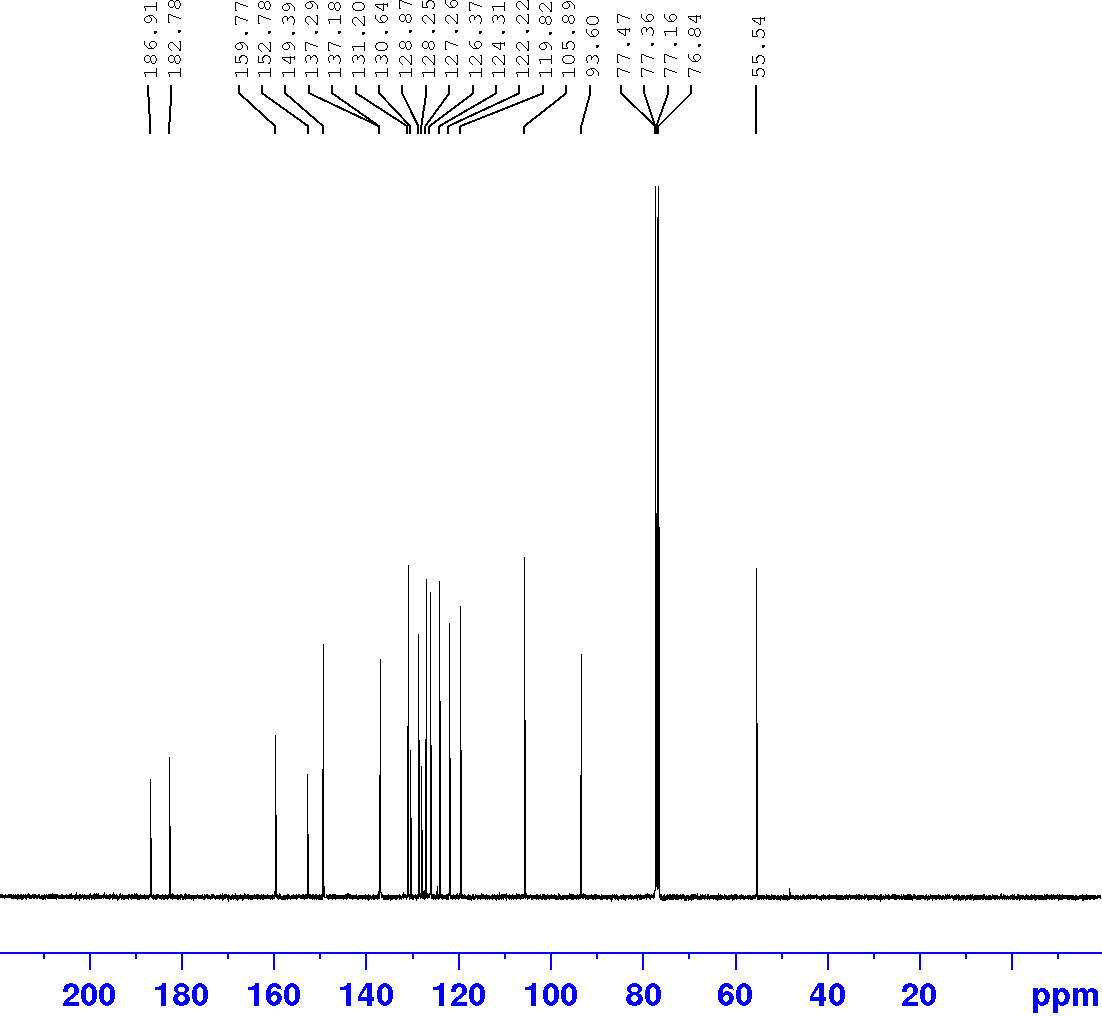


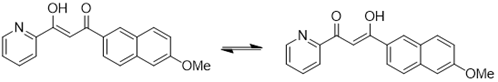


**Figure A2.29:** ^13^C NMR spectrum of B_5_ in deacidified CDCl_3_.

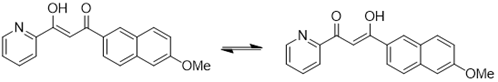


**Figure A2.30:** HRMS spectrum of B_5._ Formation of the product was confirmed by the appearance of peak at 306.11220 which agrees the calculated value.


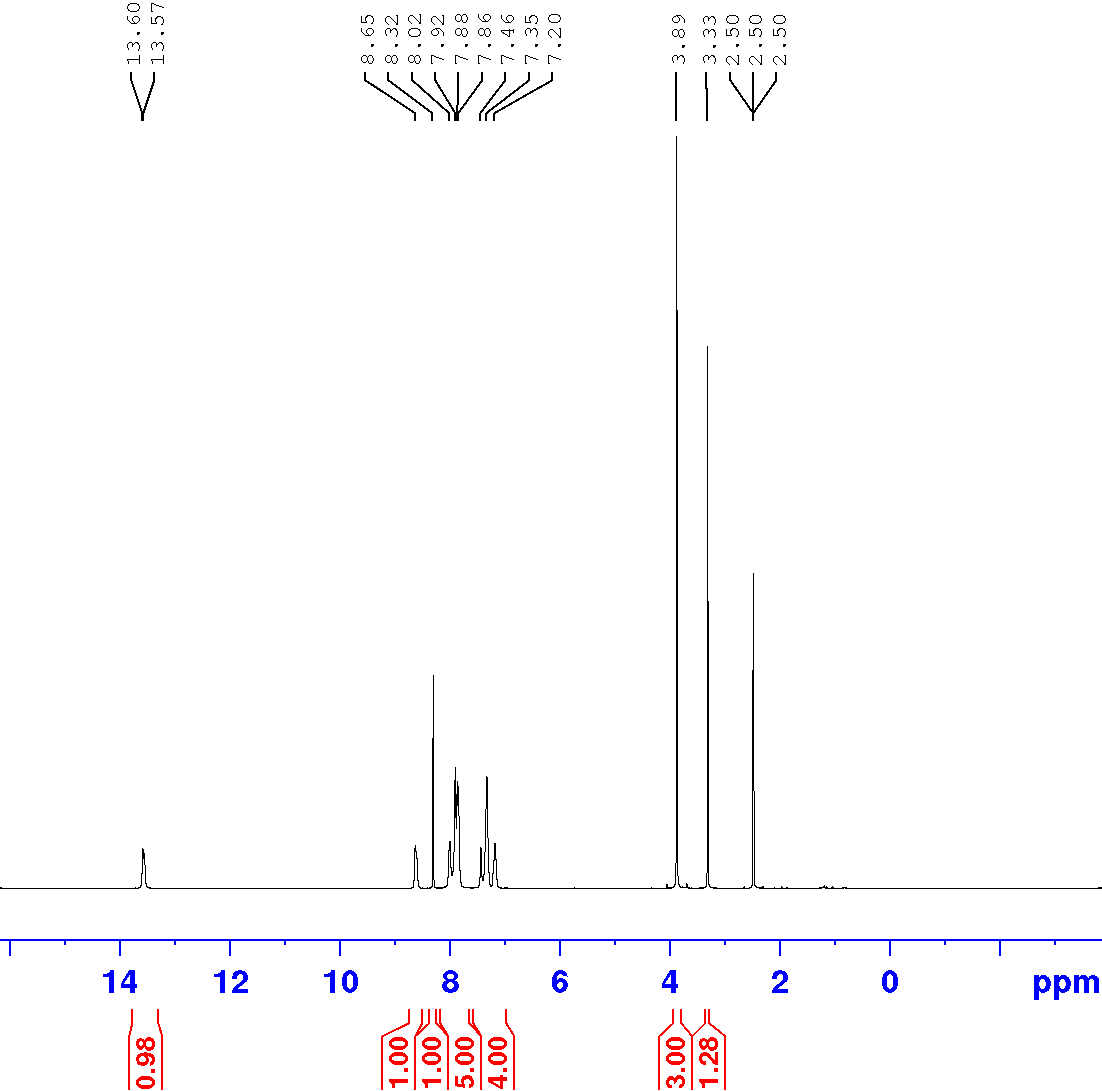


-OCH_3_ proton


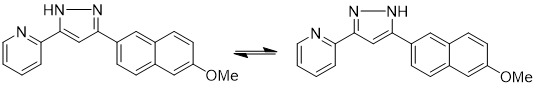


-NH proton of pyrazole

**Figure A2.31: ^1^**H NMR spectrum of C_5_ in DMSO-*d*_6._ Formation of the product was confirmed by the disappearance of enolic proton, appearance of -NH proton and upfield shifting of the peaks due to replacement of oxygen with nitrogen.


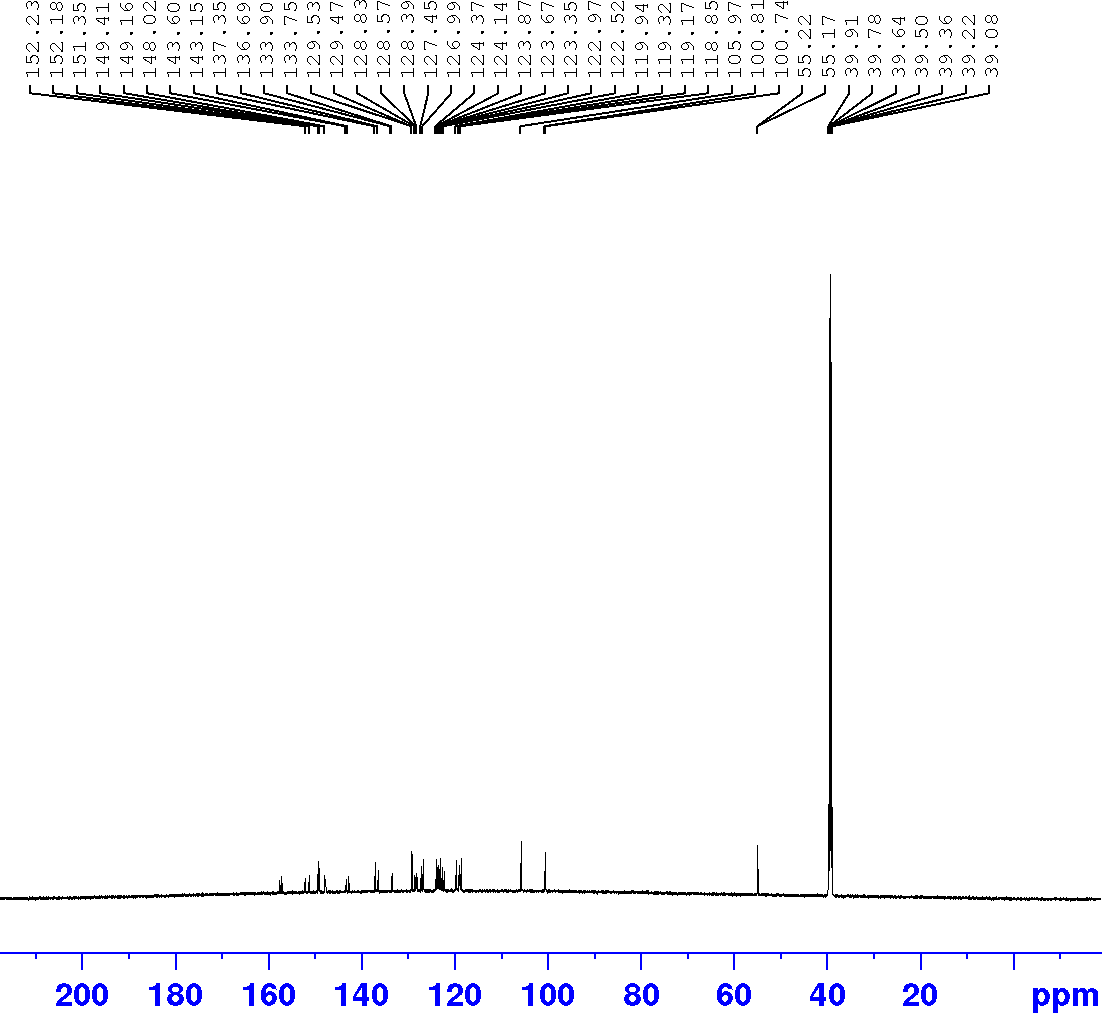


**
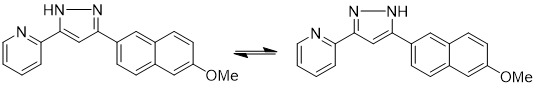
**

**Figure A2.32:** ^13^C NMR spectrum of C_5_ DMSO-*d*_6._

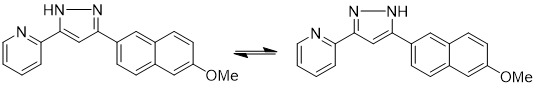


**Figure A2.33:** HRMS spectrum of C_5._ Formation of the product was confirmed by the peak at 302.12852 which agrees the calculated value.


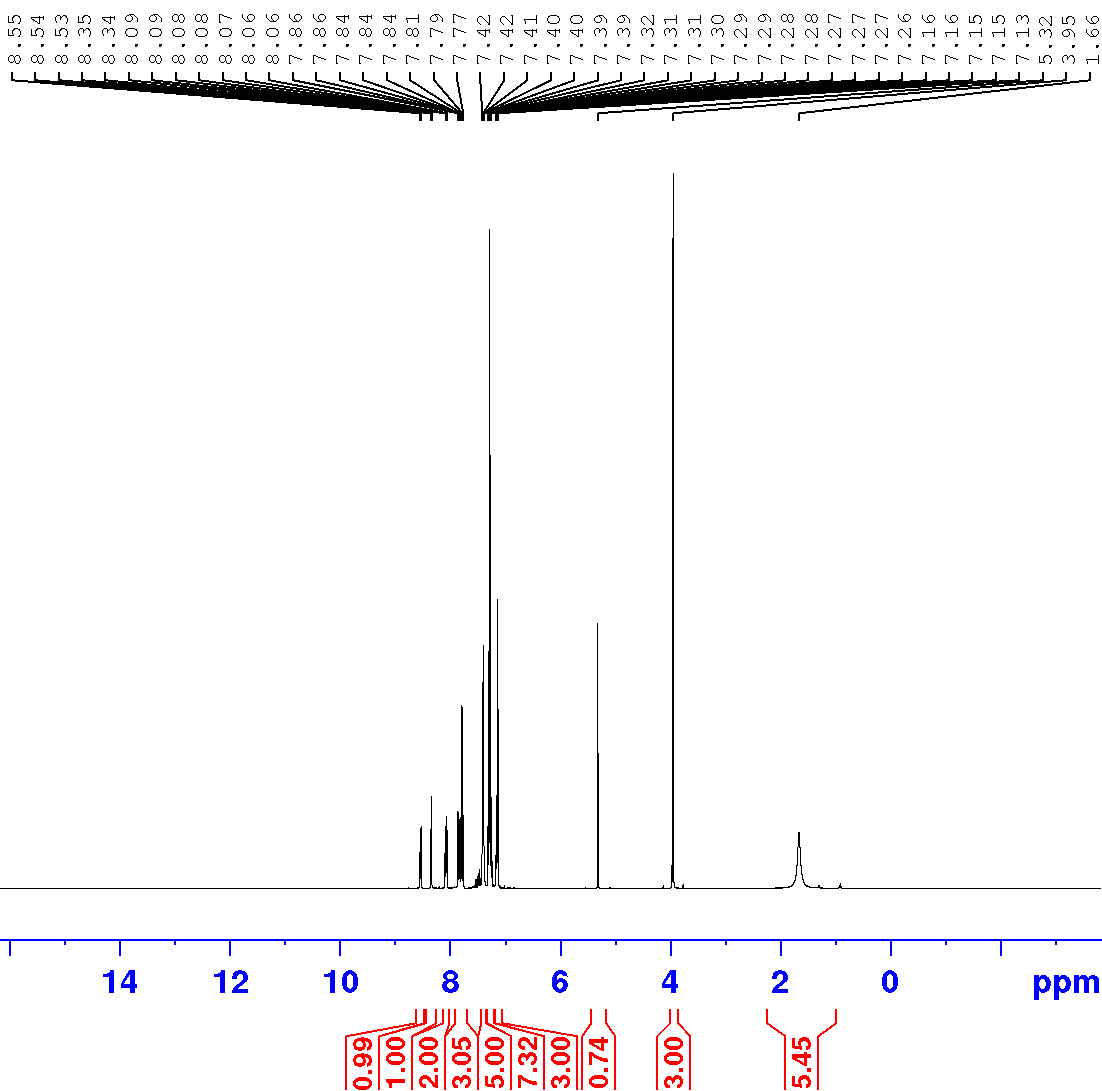


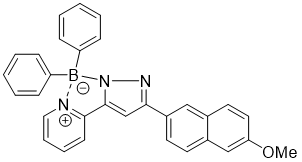


**Figure A2.34:** ^1^H NMR spectrum of P_5_ in deacified CDCl_3_. Formation of the product was confirmed by the disappearance of -NH proton and upfield shifting of the protons due to formation of electron rich molecule.


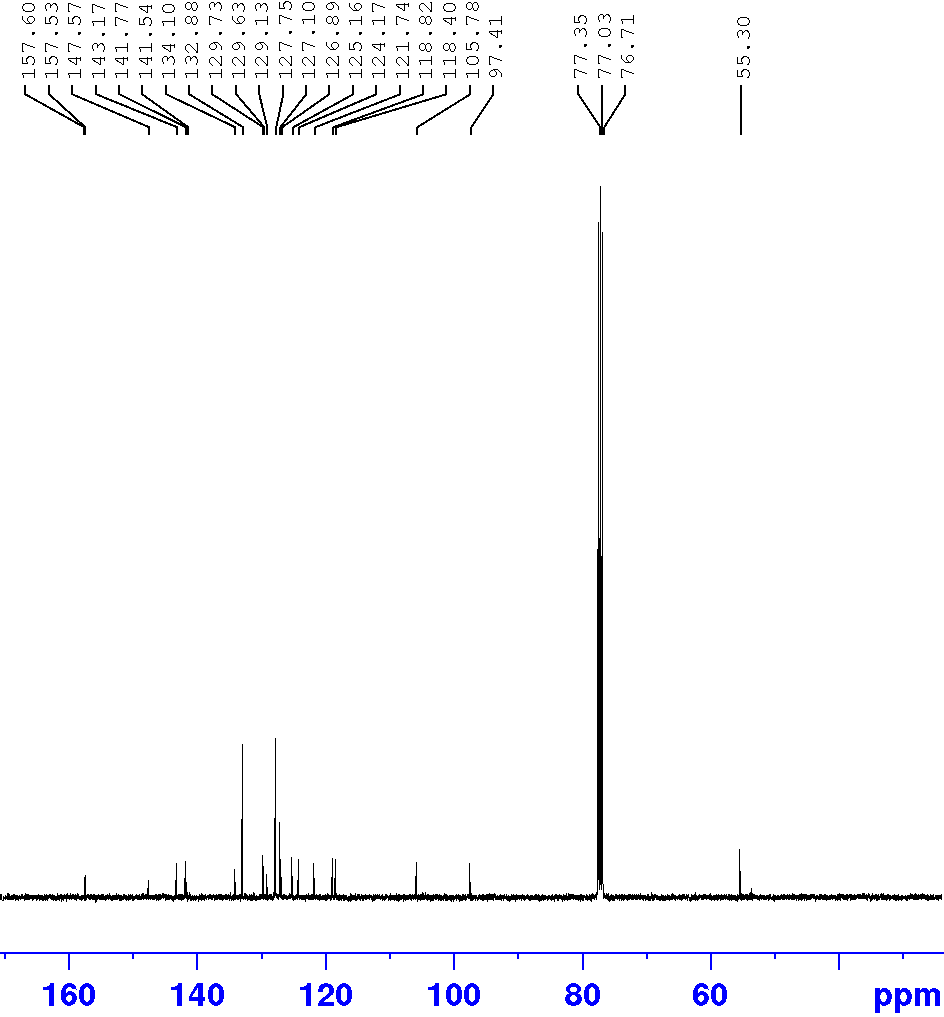


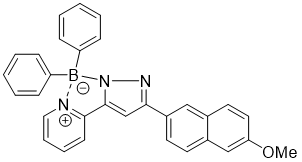


**Figure A2.35:** ^13^C NMR spectrum of P_5_ in deacidified CDCl_3_~~.~~

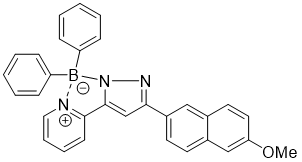


**Figure A2.36:** HRMS spectrum of P_5._ Formation of the product was confirmed by the appearance of the peak at 466.2099 which agrees the calculated value.


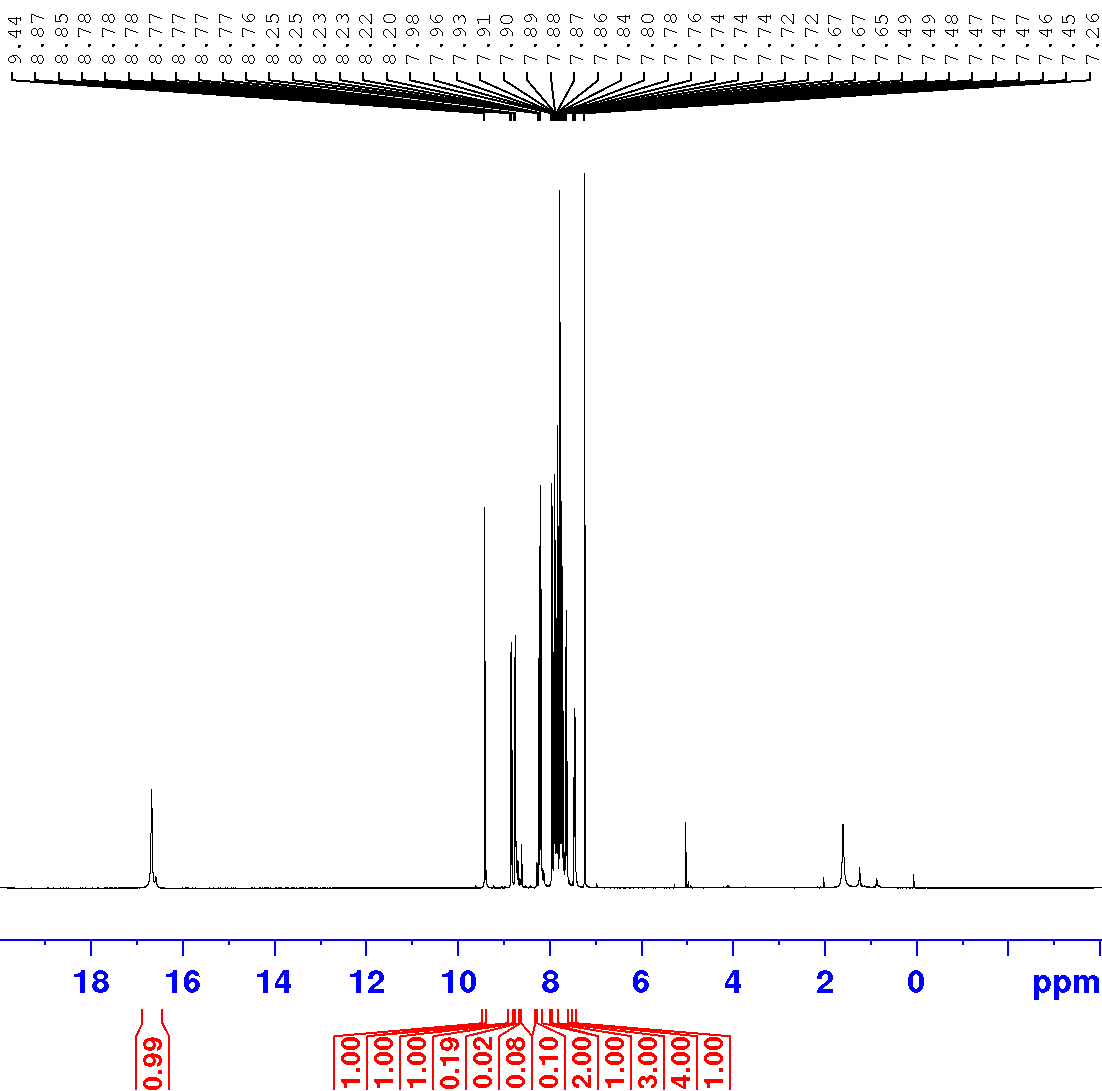

-OH proton of enol

**Figure A2.46:** ^1^H NMR spectrum of B_6_ in deacidified CDCl_3_. Formation of the product was confirmed by the appearance of the characteristic enolic proton.


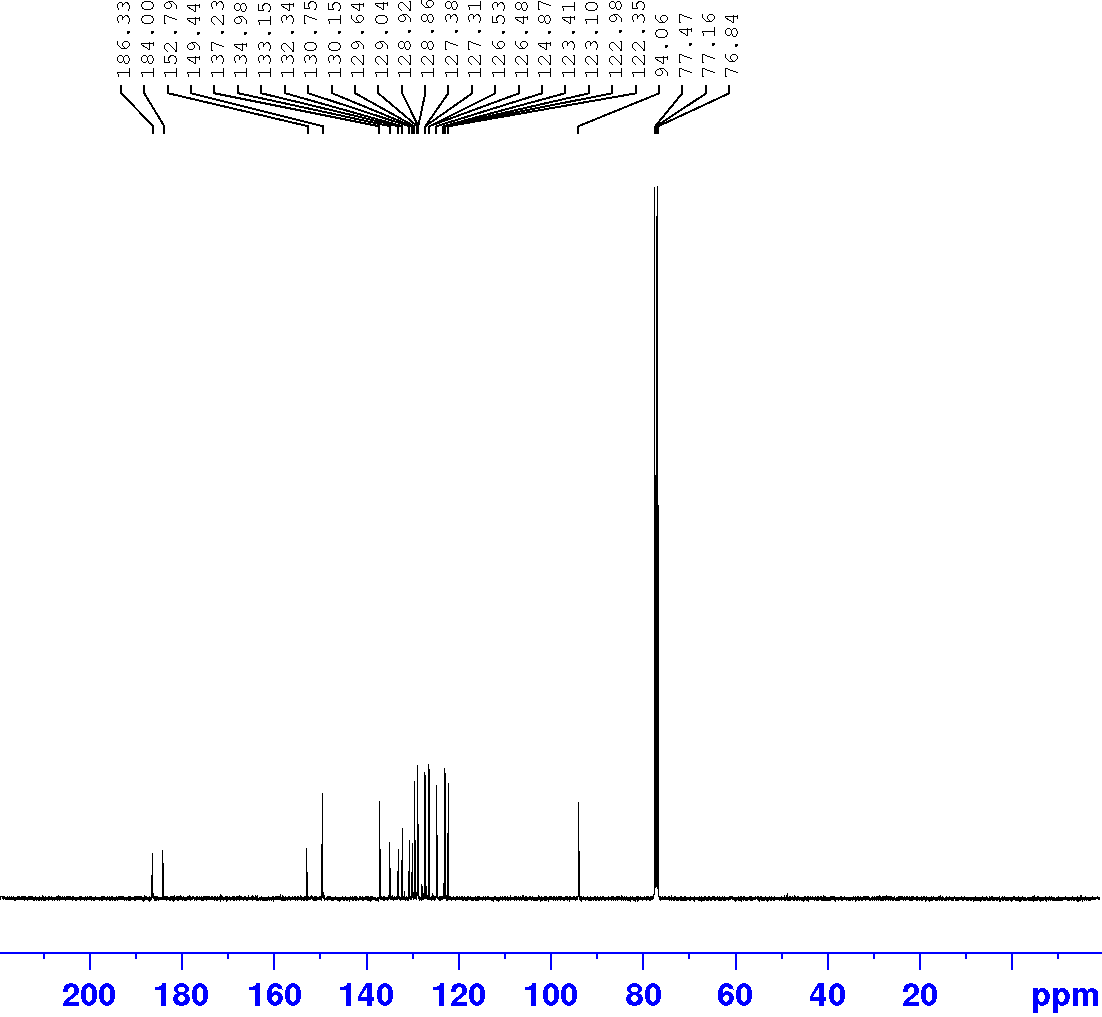

**Figure A2.47:** ^13^C NMR spectrum of B_6_ in deacidified CDCl_3_.

**Figure A2.48:** ^1^HRMS spectrum of B_6.._Formation of the product was confirmed by the peak at 326.11734 which agrees the calculated value.


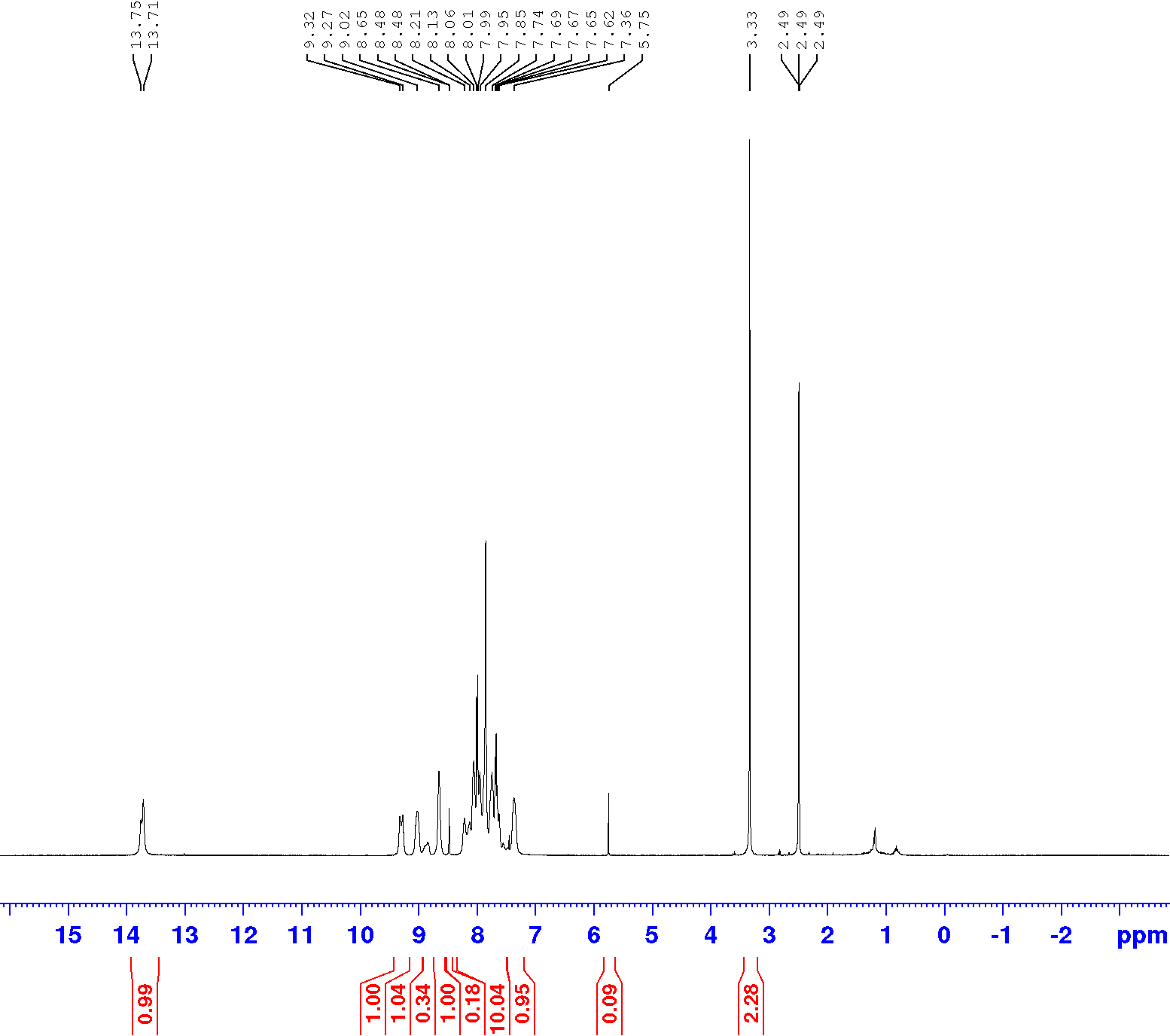


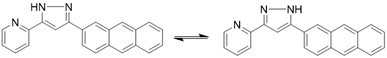


-NH proton of pyrazole

**Figure A2.49:** ^1^H NMR spectrum of C_6_ in DMSO-*d*_6._ Formation of the product was confirmed by the disappearance of enolic protons, appearance of -NH protons and upfield shifting of the peaks due to replacement of oxygen with nitrogen.


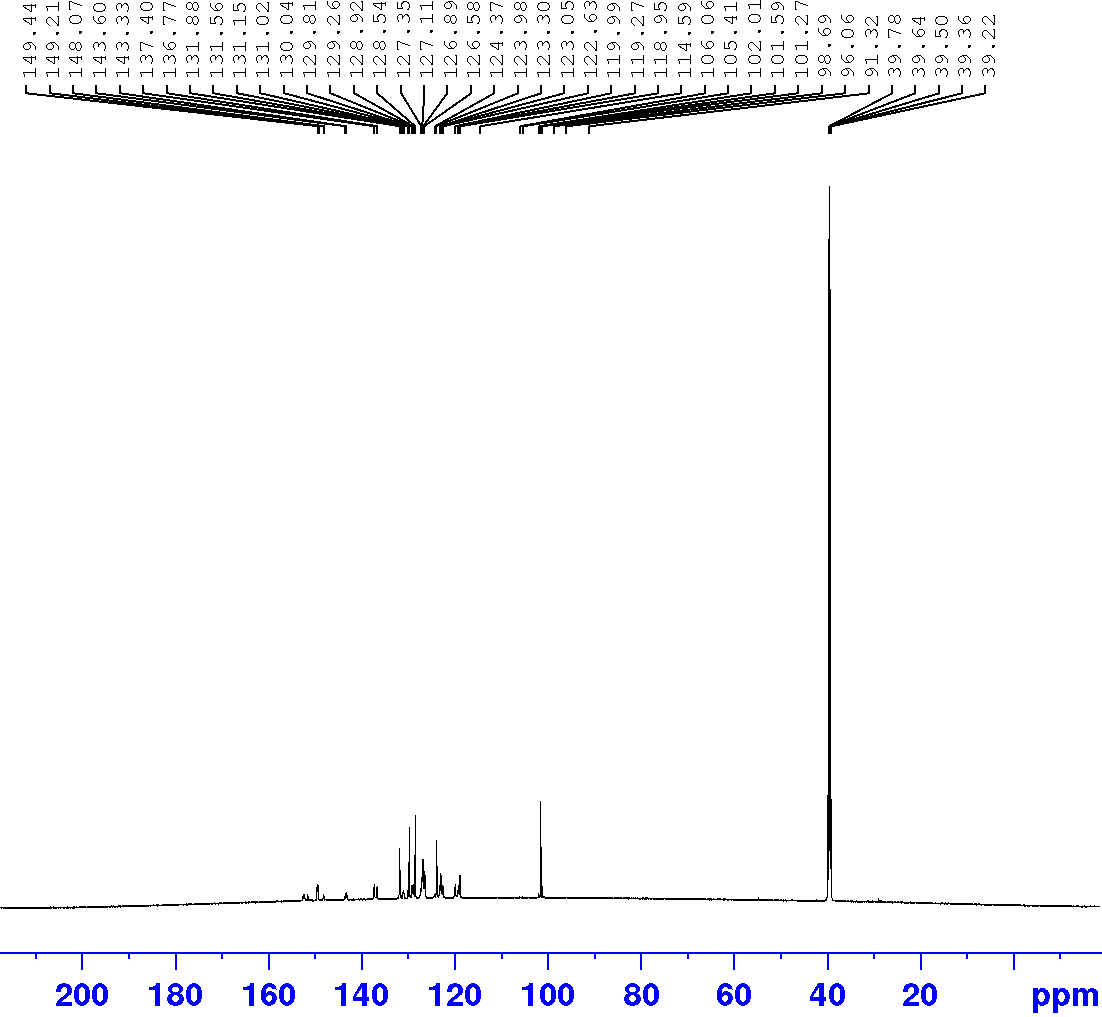


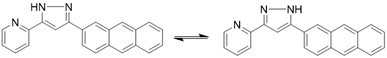


**Figure A2.50:** ^13^C NMR spectrum of C_6_ in DMSO-*d*_6._

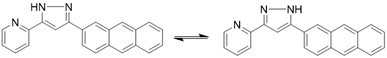


**Figure A2.51:** HRMS spectrum of C_6._ Formation of the product was confirmed by the peak at 322.13362 which agrees the calculated value.


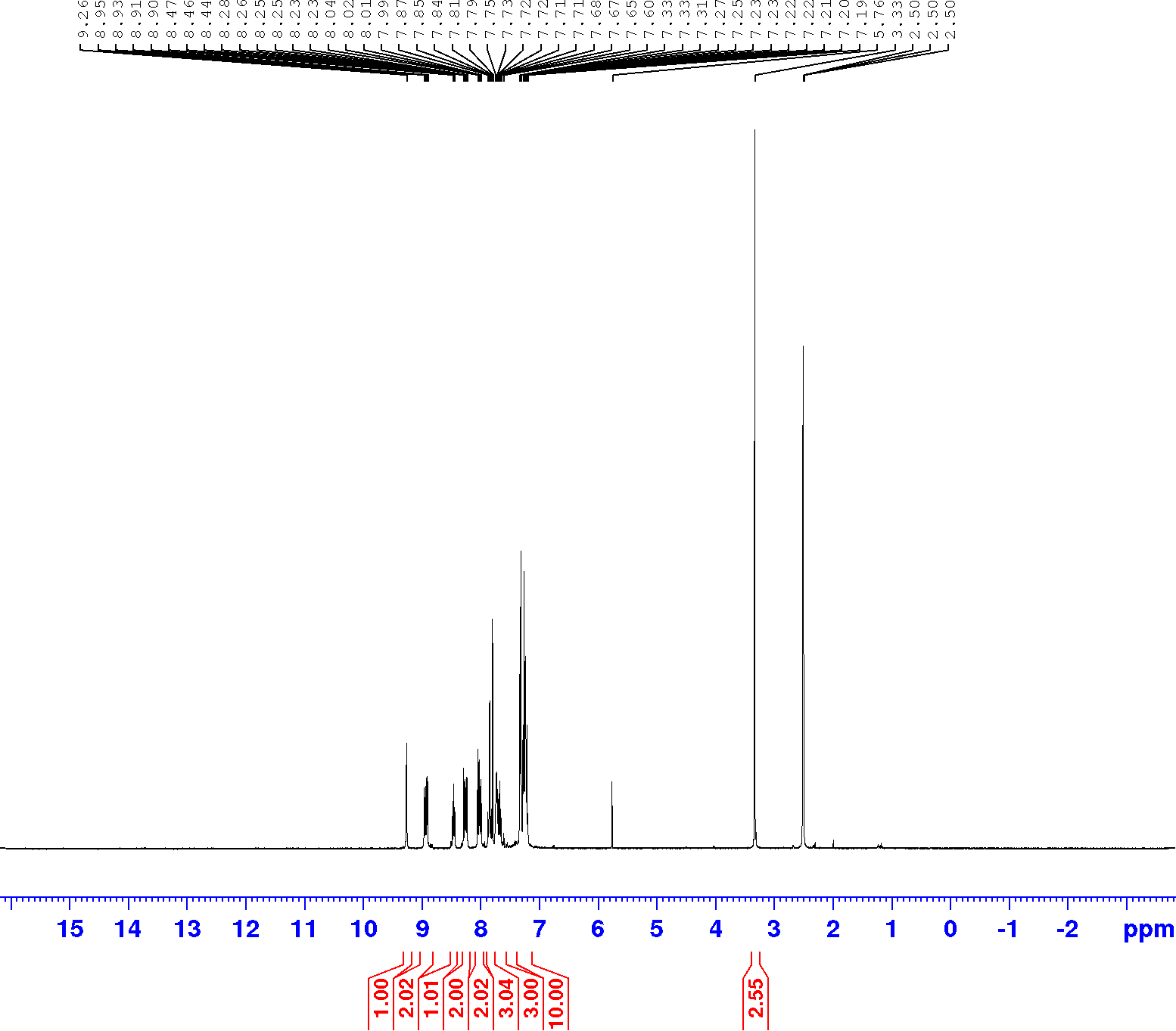


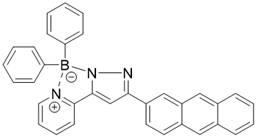


**Figure A2.52:** ^1^H NMR spectrum of C_6_ in DMSO-*d*_6._ Formation of the product was confirmed by the disappearance of the -NH proton and upfield shifting of the peaks due to formation of electron rich molecule.


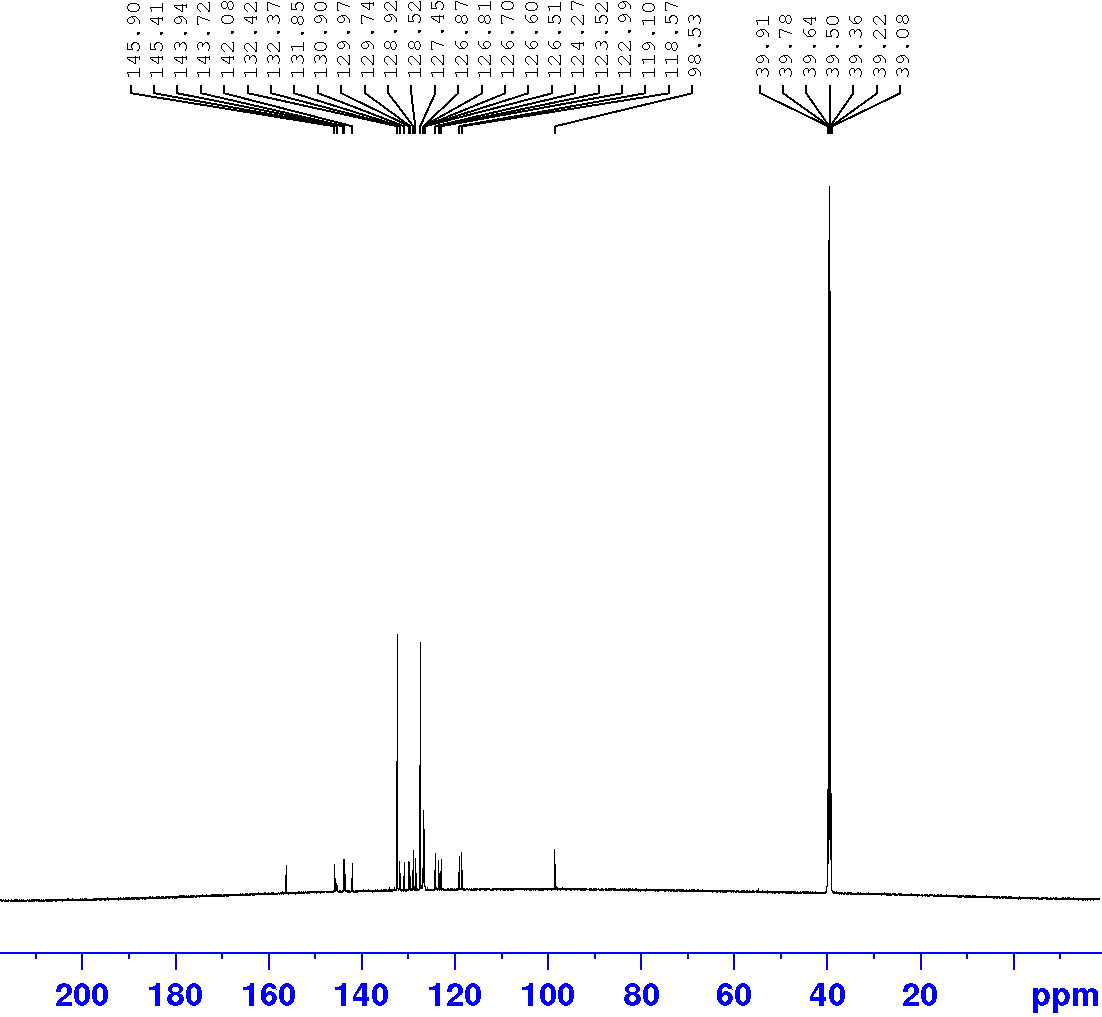


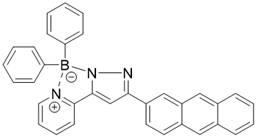


**Figure A2.53:** ^13^C NMR spectrum of C_6_ in DMSO-*d*_6._

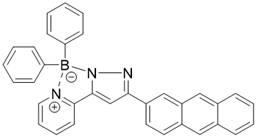


**Figure A2.54:** HRMS spectrum of C_6._ Formation of the product was confirmed by the peak at 486.21337 which agrees the calculated value.

# Figures

*
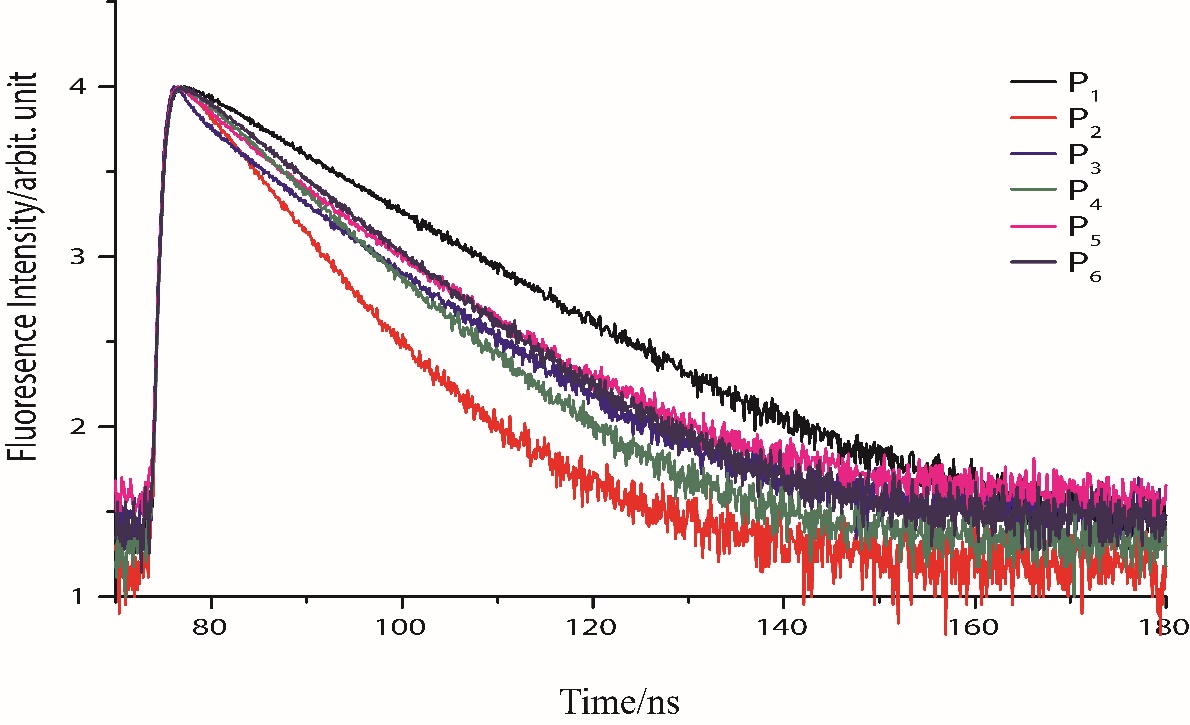
*

**Figure S1:** Time-resolved fluorescence spectrum of P_1_-P_6_ in CH_2_Cl_2_.


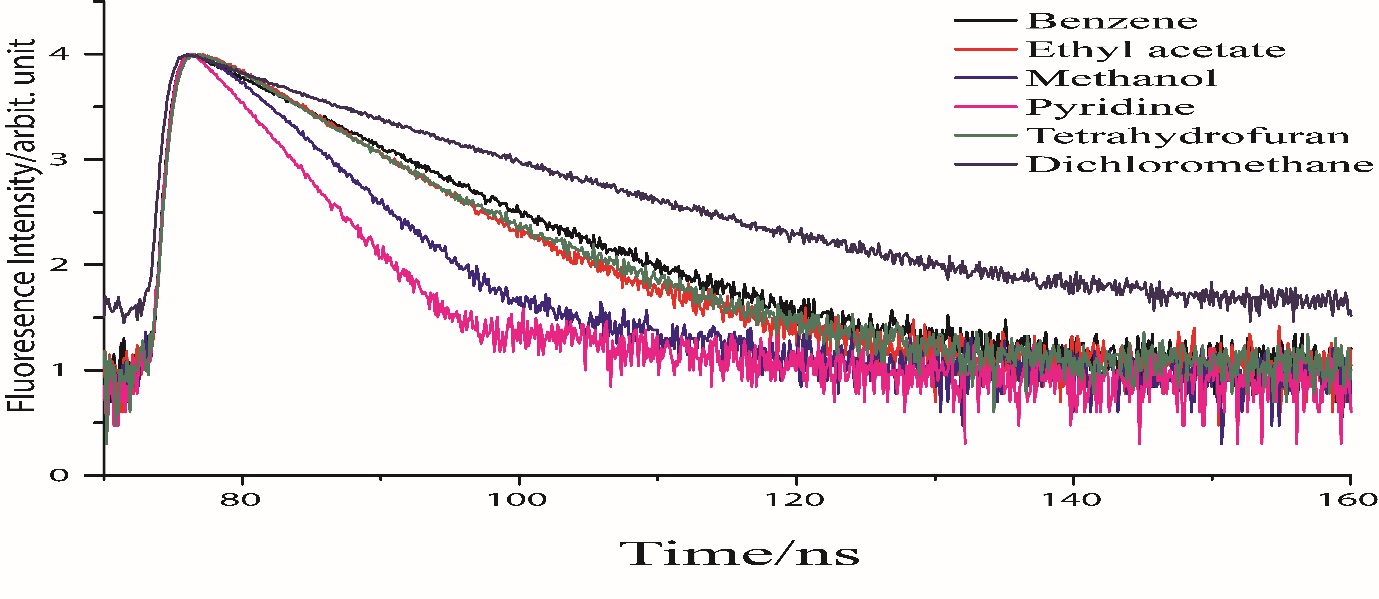


**Figure S2:** Time-resolved fluorescence spectrum of P_5_ in various organic solvents.

# References

1. Fulmer, G. R.; Miller, A. J. M.; Sherden, N. H.; Gottlieb, H. E.; Nudelman, A.; Stoltz, B. M.; Bercaw, J. E.; Goldberg, K. I., *Organometallics* **2010,** *29* (9), 2176-2179.
